# Supplementary material for: ADP-ribosylation of NuMA promotes DNA single-strand break repair and transcription
Source: Cell Rep. 2025 May 20;44(6):115737. doi: 10.1016/j.celrep.2025.115737 (PMC12187637; doi:10.1016/j.celrep.2025.115737)
Supplement: Document S1. Figures S1–S8 and Tables S1–S3 [file mmc1.pdf]

Cell Reports, Volume *44*

Supplemental information

ADP-ribosylation of NuMA promotes DNA single-strand break repair and transcription

Arwa A. Abugable, Chunyan Liao, Sarah Antar, Matthew Dowson, and Sherif F. El-Khamisy

ADP-ribosylation of NuMA promotes DNA single-strand break repair and transcription

Arwa A. Abugable[1,2,5], Chunyan Liao[1,2], Sarah Antar[1,2,3], Matthew Dowson[1,2], Sherif F. El-Khamisy[1,2,4,6,*]
[1]School of Biosciences, Firth Court, University of Sheffield, Sheffield, UK
[2]The Healthy Lifespan and Neuroscience Institutes, University of Sheffield, Sheffield, UK
[3]Medical Biochemistry and Molecular Biology Department, Faculty of Medicine, Mansoura University, Egypt
[4]Institute of Cancer Therapeutics, School of Pharmacy and Medical Sciences, University of Bradford, Bradford, UK
[5]Present address: Department of Biochemistry, University of Oxford, South Parks Road, Oxford, UK
[6]Lead Contact
*Correspondence: s.el-khamisy@bradford.ac.uk

Table of Contents

Figure S1: AlphaFold modelling of NuMA^{WT} and NuMA^{PARmut} (related to Figure 2) 2

Figure S2: Corrected mutation in NuMA^{WT} does not impair SSBR kinetics (related to Figure 4a, b) 3

Figure S3: Enrichment of NuMA at specific genomic loci (related to Figures 4c,d) 4

Figure S4: Transcript levels of NuMA-regulated genes (related to Figure 5) 4

Figure S5: Cold shock does not change transcript levels of NuMA-regulated genes (related to Figure 5) 5

Figure S6: Enrichment of RNAPII at specific genomic loci (related to Figure 5) 5

Figure S7: TDP1 over-expression does not rescue repair and transcription defect of NuMA^{PARmut} (related to Figures 4 and 5) 6

Figure S8: Uncropped blots (Related to Figures 1, 3 and S2) 7

Table S1: ADP-ribosylation sites that are mutated in NuMA^{PARmut} (related to Figure 2) 8

Table S2: List of primers used in this study 8

Table S3: List of siRNA sequences used in this study 10

References 10

Figure S1: AlphaFold modelling of NuMA^{WT} and NuMA^{PARmut} (related to Figure 2)

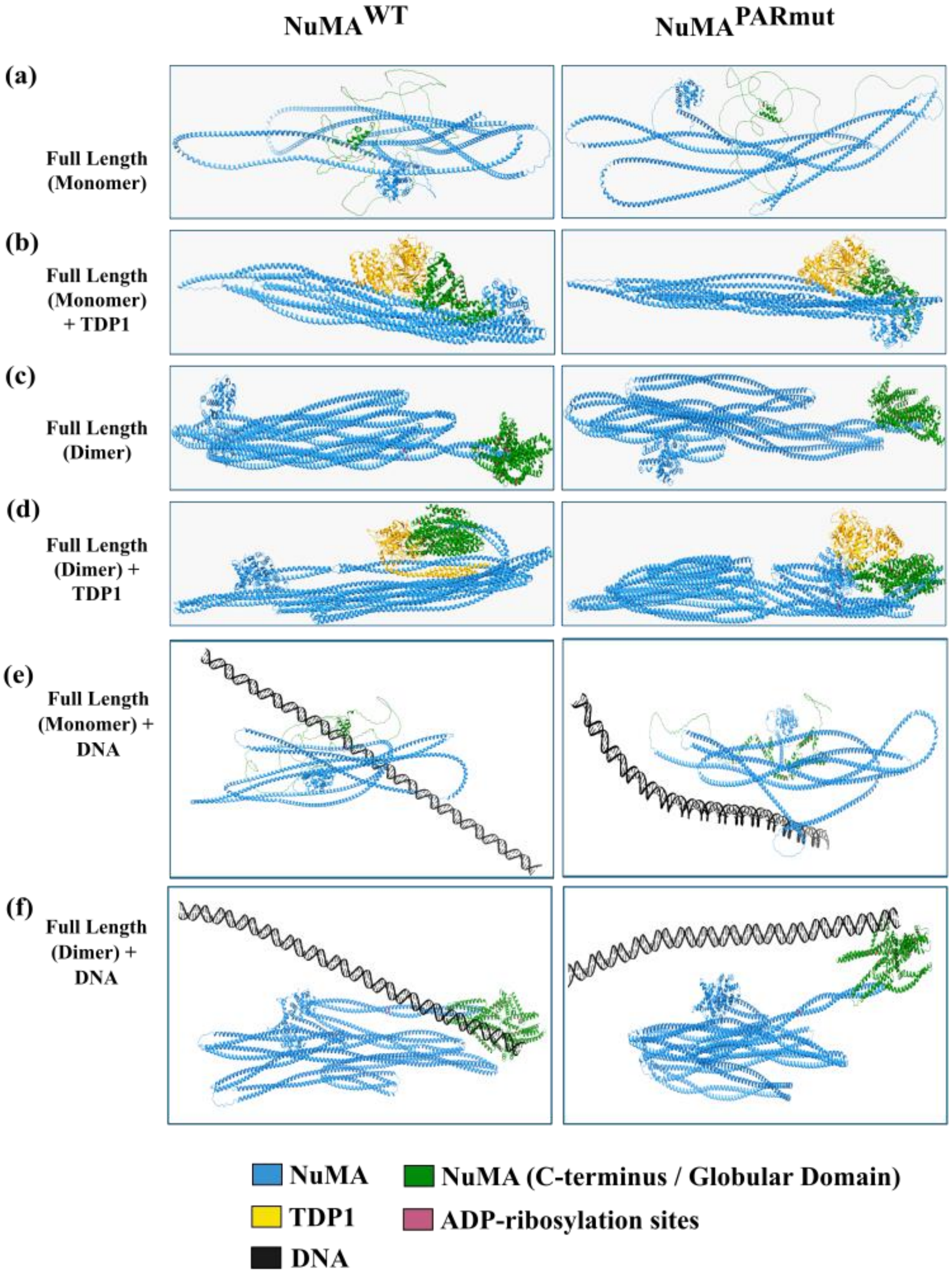

Figure S1: AlphaFold modelling of NuMA^{WT} and NuMA^{PARmut}

Predicted structures of (**a**) full length NuMA^{WT} and NuMA^{PARmut} as monomers and (**b**) in complex with TDP1, (**c**) full length NuMA^{WT} and NuMA^{PARmut} as dimers and (**d**) in complex with TDP1, (**e**) full length NuMA^{WT} and NuMA^{PARmut} as monomers in complex with DNA and (**f**) as dimers in complex with DNA.

Figure S2: Corrected mutation in NuMA^{WT} does not impair SSBR kinetics (related to Figure 4a, b)

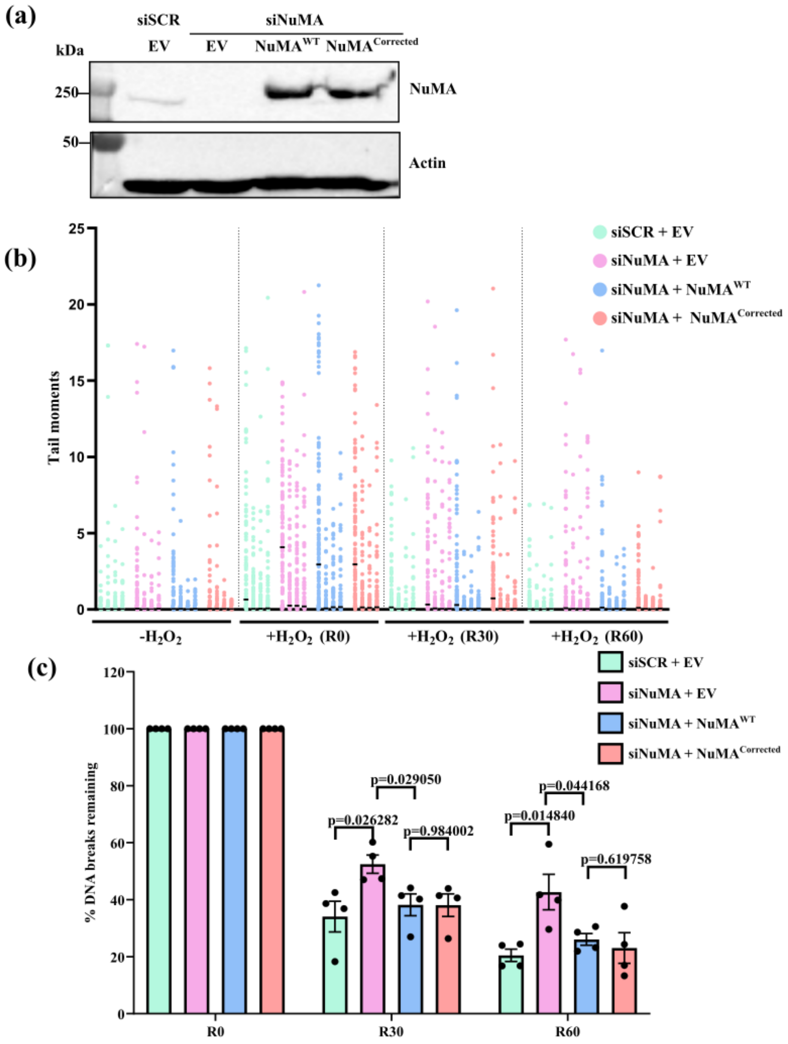

Figure S2: Corrected mutation in NuMA^{WT} does not impair SSBR kinetics

(**a**) Representative immunoblotting from whole cell extracts showing the successful knock down of endogenous NuMA with siRNA and transfection of the empty vector (EV), NuMA^{WT} and NuMA^{Corrected} constructs. (**b and c**) Cells were transfected with siSCR or siNuMA and then complemented with either EV, NuMA^{WT} or NuMA^{Corrected} constructs. The cells were left untreated or treated with 10 µM H_2O_2 for 10 minutes on ice in the dark, followed by recovery in H_2O_2-free media for 0, 30 and 60 minutes, denoted as R0, R30 and R60, respectively, before being subjected to alkaline comet assay. (**b**) Violin plot showing the distribution of the of comet tail moments at the indicated time points. The data shown are from 4 biological replicates. (**c**) Bar plot showing the % DNA breaks remaining during the recovery time points. The bar char represents data from 4 biological replicates with error bars representing the standard error of the mean. Two-sided unpaired Student t-test was conducted.

Figure S3: Enrichment of NuMA at specific genomic loci (related to Figures 4c,d)

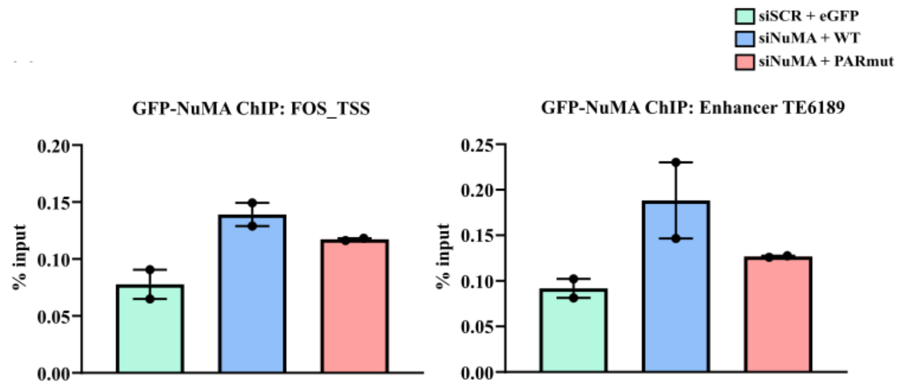

Figure S3: Enrichment of NuMA at specific genomic loci

Cells were transfected with siSCR or siNuMA and then complemented with either empty vector (EV), $NuMA^{WT}$ or $NuMA^{PARmut}$ constructs, serum-starved for 48 hours and then treated with 10 μM H_2O_2 for 10 minutes on ice in the dark, followed by a 90-minute recovery in serum-containing media. GFP-NuMA ChIP-qPCR was conducted at the *FOS* promoter and TE6189 enhancer. The bar chart shows the % input from 2 biological replicates with error bars representing the range.

Figure S4: Transcript levels of NuMA-regulated genes (related to Figure 5)

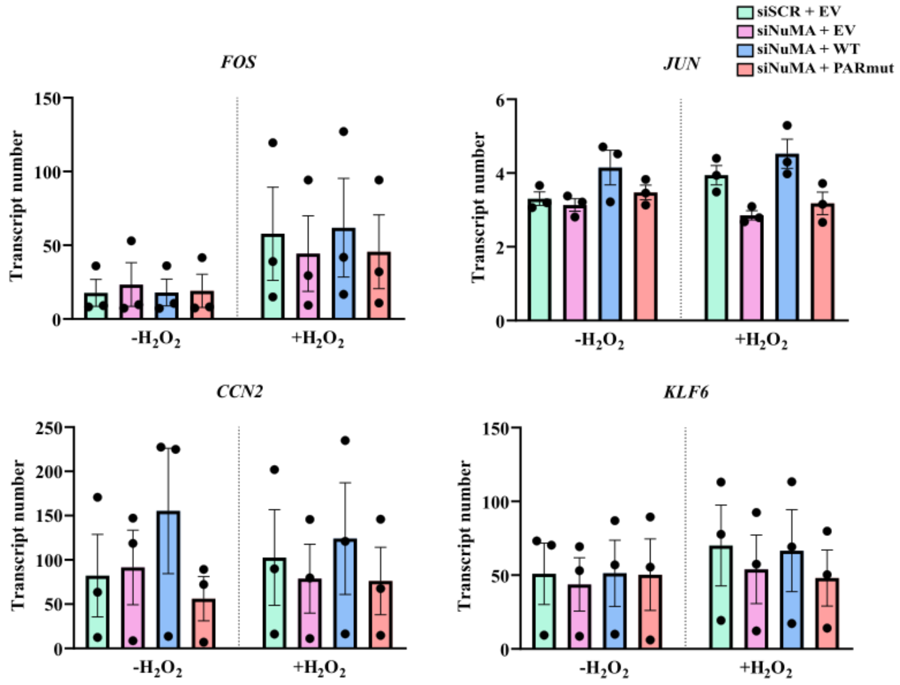

Figure S4: Transcript levels of NuMA-regulated genes

Cells were transfected with siSCR or siNuMA, complemented with EV, $NuMA^{WT}$ or $NuMA^{PARmut}$, serum starved for 48 hours, treated with 10 μM H_2O_2 for 10 minutes on ice in the dark and recovered in serum-containing media for 90 minutes. Bar plots showing transcript number following qPCR of *FOS*, *JUN*, *CCN2* and *KLF6* (n=3 biological replicates, mean±SEM).

Figure S5: Cold shock does not change transcript levels of NuMA-regulated genes (related to Figure 5)

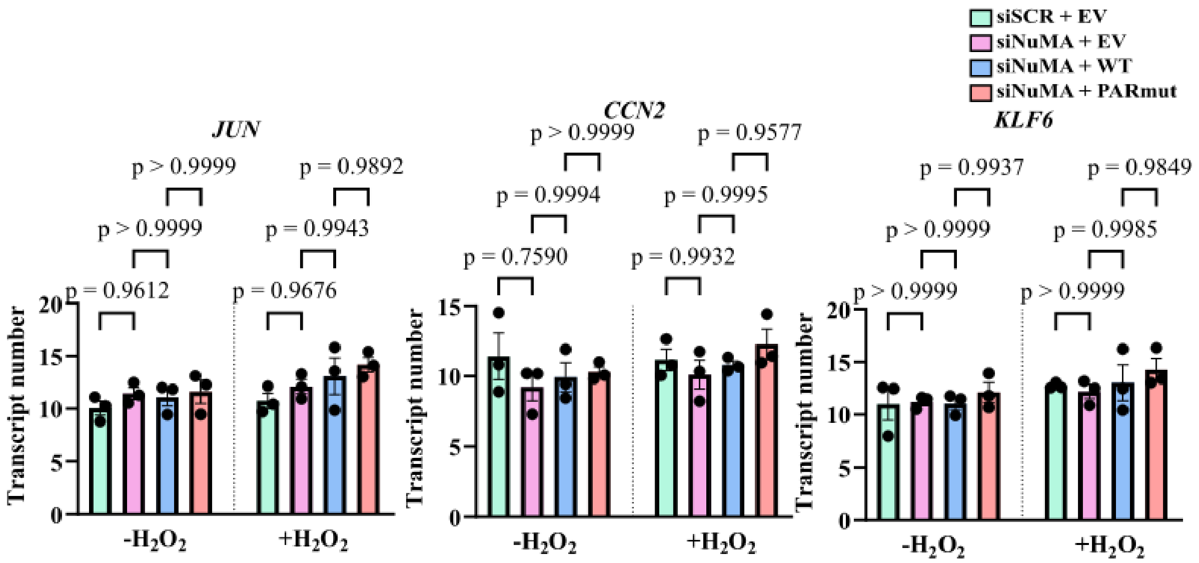

Figure S5: Cold shock does not change transcript levels of NuMA-regulated genes

Cells were transfected with siSCR or siNuMA and then complemented with either empty vector (EV), $NuMA^{WT}$ or $NuMA^{PARmut}$ constructs, serum-starved for 48 hours and then treated with 10 µM H_2O_2 in cold PBS (+H_2O_2) or just cold PBS (-H_2O_2) for 10 minutes on ice in the dark, followed by qPCR analysis for the expression of *JUN*, *CCN2* and *KLF6*. Bar plots showing transcript number (n=3 biological replicates, mean±SEM). One-way ANOVA was conducted.

Figure S6: Enrichment of RNAPII at specific genomic loci (related to Figure 5)

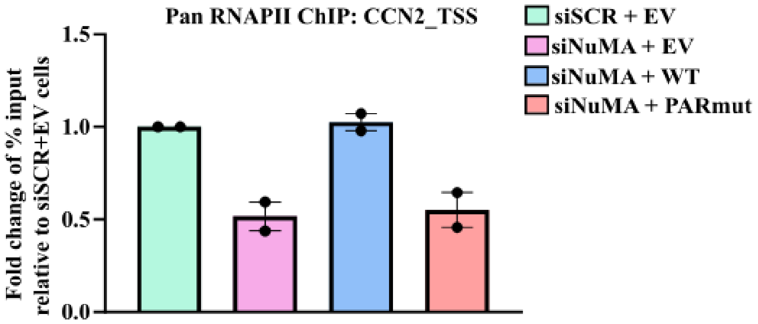

Figure S6: Enrichment of RNAPII at CCN2 promoter

Cells were transfected with siSCR or siNuMA and then complemented with either empty vector (EV), $NuMA^{WT}$ or $NuMA^{PARmut}$ constructs, serum-starved for 48 hours and then treated with 10 µM H_2O_2 for 10 minutes on ice in the dark, followed by a 90-minute recovery in serum-containing media. Pan RNAPII ChIP-qPCR was conducted at the *CCN2* promoter. The bar chart shows the fold change in % input relative to the siSCR+EV-transfected cells and represents data from 2 biological replicates with error bars representing the range.

Figure S7: TDP1 over-expression does not rescue repair and transcription defect of NuMA^{PARmut} (related to Figures 4 and 5)

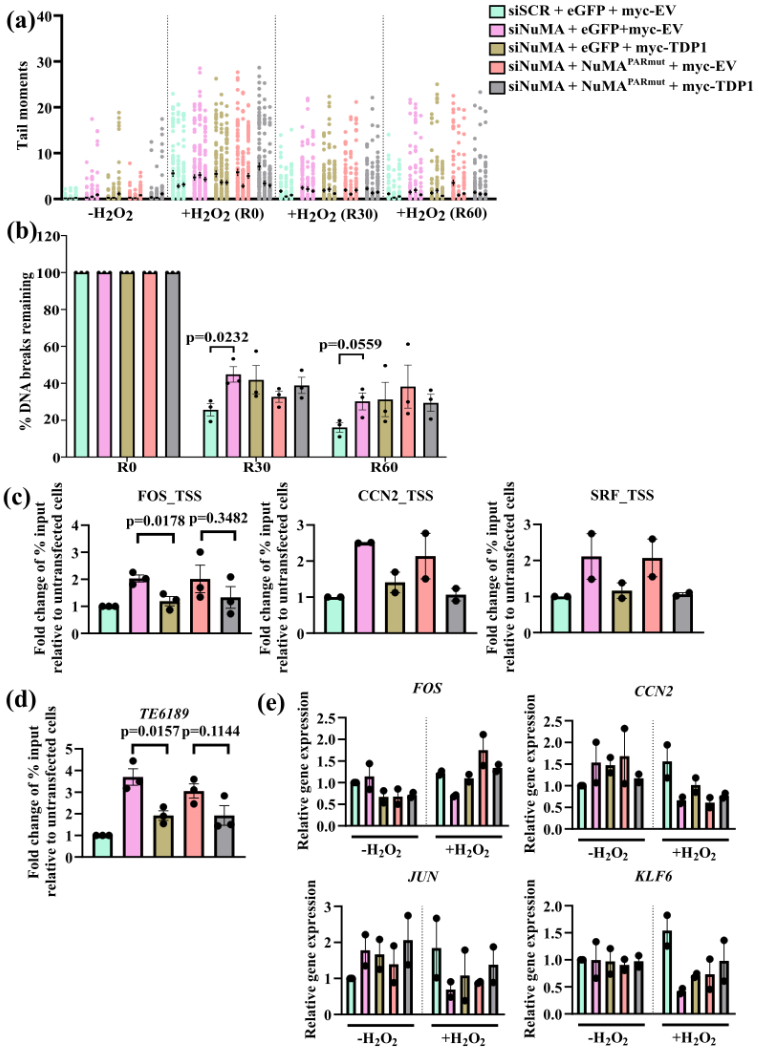

Figure S7: TDP1 over-expression does not rescue repair and transcription defect of NuMA^{PARmut}

(a-d) Cells transfected with siSCR or siNuMA and then complemented with either EV, NuMA^{WT} or NuMA^{PARmut}, and either myc-EV or myc-TDP1. (**a and b**) Cells were left untreated or treated with 20 µM H_2O_2 for 10 minutes on ice in the dark, followed by recovery in H_2O_2-free media for 0, 30 and 60 minutes (R0, R30 and R60) and used for alkaline comet assay. (**a**) Scatter plot of comet tail moments at the indicated time points. (**b**) Bar plot of % DNA breaks remaining (n=3 biological replicates, mean±SEM). Two-sided unpaired Student t-test was conducted. (**c-d**) OGG1-AP-qPCR at the (**c**) *FOS*, *CCN2*, *SRF* promoters and (**d**) *TE6189* enhancer. Bar charts show fold change in % input relative to the untransfected cells (n=3 biological replicates, mean±SEM for *FOS* promoter and *TE6189*; n=2 biological replicates, range for *CCN2* and *SRF* promoters). Two-sided unpaired Student t-test was conducted. (**e**) Transfected cells were serum starved for 48 hours, then left untreated or treated with 10 µM H_2O_2 for 10 minutes on ice in the dark and recovered in serum-containing media for 90 minutes. Transcript levels were measured by qPCR. Expression in untreated and siSCR H_2O_2-treated cells were normalised to siSCR untreated cells, while all other conditions were normalised to siSCR H_2O_2-treated cells (n=2 biological replicates, range).

Figure S8: Uncropped blots (Related to Figures 1, 3 and S2)

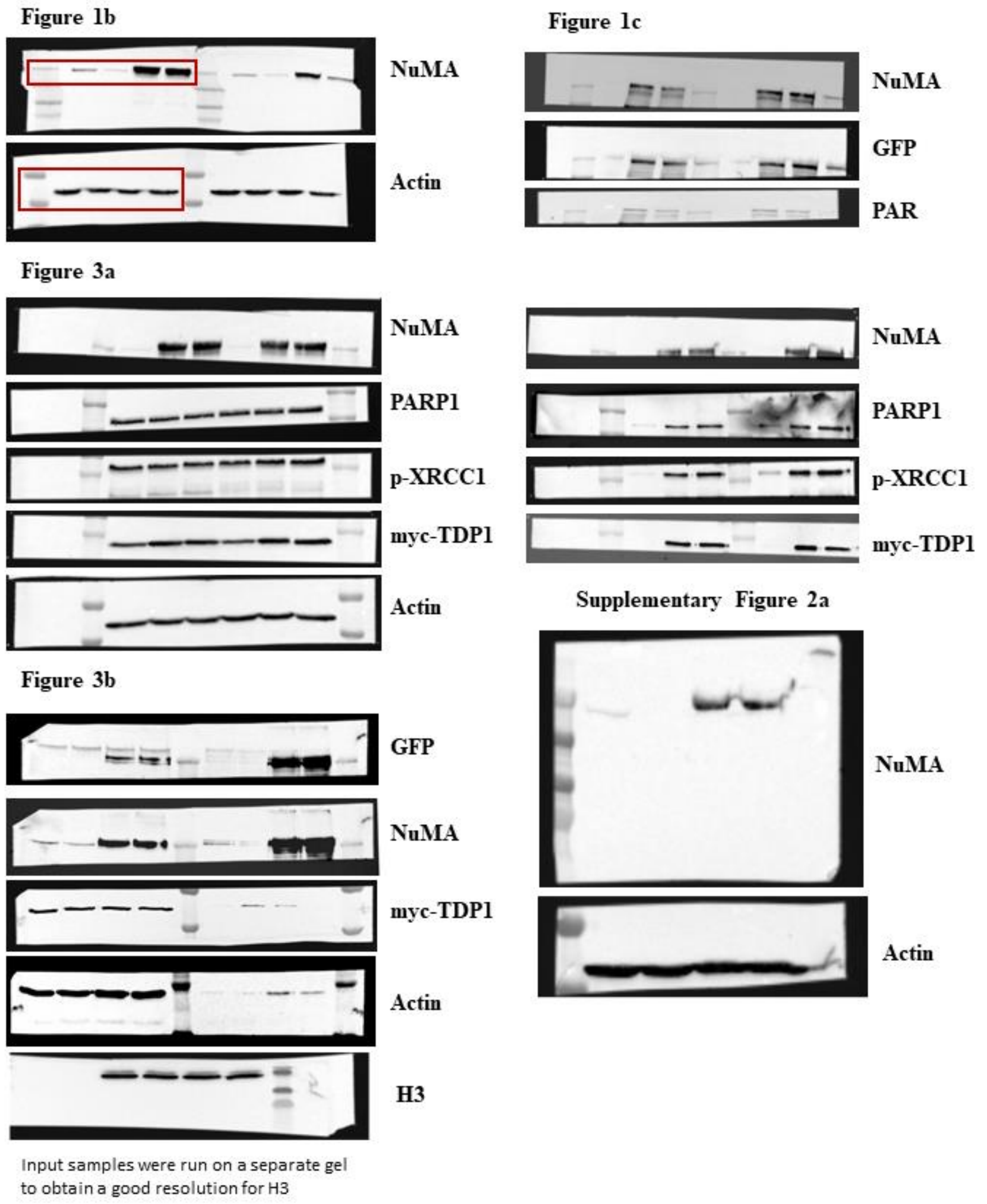

Figure S8: Uncropped blots

Uncropped images of the blots shown in the manuscript.

Table S1: ADP-ribosylation sites that are mutated in NuMA^{PARmut} (related to Figure 2)

Table S1: Amino acid positions that have been mutated in the WT construct to generate the PARmut construct.

Amino Acid Position	Amino acid in NuMA^{WT}	Amino acid in NuMA^{PARmut}
**1609**	H	A
**1806**	S	A
**1840**	S	A
**1852**	S	A
**1887**	S	A
**1892**	S	A
**1969**	S	A
**1991**	S	A
**2003**	S	A
**2008**	S	A
**2009**	C	A
**2047**	S	A
**2051**	S	A
**2062**	S	A
**2069**	S	A
**2082**	S	A

Table S2: List of primers used in this study

Table S2: Sequences of the primers and DNA block used in this study

Primer name	Primer sequence	Purpose	Reference
**1609_Fwd**	CAGAAGGAGCAGGCAGCTGAGGCCTA TAAGCTGCAGATGGAG	Generation of NuMA^{PARmut}	This study
**1609_Rev**	CTCCATCTGCAGCTTATAGGCCTCAGC TGCCTGCTCCTTCTG	Generation of NuMA^{PARmut}	This study
**2082_Fwd**	GGCTTCCCCCAACACTCGCGCTGGAA CCCGCCGTTCTCCG	Generation of NuMA^{PARmut}	This study
**2082_Rev**	CGGAGAACGGCGGGTTCCAGCGCGAG TGTTGGGGGAAGCC	Generation of NuMA^{PARmut}	This study
**AF**	TCCAAGAGTTGGCAGCCGTACG	Generation of NuMA^{PARmut}	This study
**AR**	GCGGGTCTTACGACCCGAGTC	Generation of NuMA^{PARmut}	This study
**BF**	GGAGACGTCTTCCTGGACTCG	Generation of NuMA^{PARmut}	This study
**BR**	TTATCTAGATCCGGTGGATCCG	Generation of NuMA^{PARmut} & NuMA^{Corrected}	This study
**DNA block**	GGAGACGTCTTCCTGGACTCGGGTCGT AAGACCCGCGCCGCTCGTCGGCGCAC CACGCAGATCATCAACATCACCATGA CCAAGAAGCTAGATGTGGAAGAGCCA GACAGCGCCAACTCATCGTTCTACAG	Generation of NuMA^{PARmut}	This study

Primer name	Primer sequence	Purpose	Reference
	CACGCGGGCTGCTCCTGCTTCCCAGGC TAGCCTGCGAGCCACCGCCTCTACTCA GTCTCTAGCTCGCCTGGGTTCTCCCGA TTATGGCAACTCAGCCCTGCTCAGCTT GCCTGGCTACCGCCCCACCACTCGCA GTTCTGCTCGTCGTGCCCAGGCCGGGG TGGCCAGTGGGGCCCCTCCAGGAAGG AACAGCTTCTACATGGGCACTTGCCA GGATGAGCCTGAGCAGCTGGATGACT GGAACCGCATTGCAGAGCTGCAGCAG CGCAATCGAGTGTGCCCCCCACATCTG AAGACCTGCTATCCCCTGGAGTCCAG GCCTTCCCTGAGCCTGGGCACCATCAC AGATGAGGAGATGAAAACTGGAGACC CCCAAGAGACCCTGCGCCGAGCCGCC ATGCAGCCAATCCAGATAGCCGAGGG CACTGGCATCACCACCCGGCAGCAGC GCAAACGGGTCGCCCTAGAGCCCCAC CAGGGCCCTGGAACTCCTGAGGCTAA GAAGGCCACCGCCGCTTTCCCACGCC CCATGACTCCCCGAGACCGACATGAA GGGCGCAAACAGAGCACTACTGAGGC CCAGAAGAAAGCAGCTCCAGCTTCTA CTAAACAGGCTGACCGGCGCCAGGCG ATGGCCTTCGCTATCCTCAACACACCC AAGAAGCTAGGGAACGCCCTTCTGCG GCGGGGAGCCGCAAAGAAGGCCCTGT CCAAGGCTTCCCCCAACACTCGCGCT		
**AF2**	TCCAAGAGTTGGCAGCCGTACG	Generation of NuMA^{Corrected}	This study
**1528_Fwd**	GAGAGGCAGCGGTTCCAGGAAGAGAG GCAGAAACTCAC	Generation of NuMA^{Corrected}	This study
**1528_Rev**	GTGAGTTTCTGCCTCTCTTCCTGGAAC CGCTGCCTCTC	Generation of NuMA^{Corrected}	This study
**FOS_TSS_Fwd**	ACACTCATTCATAAAACGCTTGT	AP-qPCR	This study
**FOS_TSS_Rev**	CTGCAGATGCGGTTGGAGTA	AP-qPCR	This study
**SRF_TSS_Fwd**	ACCATATAAGGAGCGGCCTCG	AP-qPCR	This study
**SRF_TSS_Rev**	GGGATCCCCCGACCCTTC	AP-qPCR	This study
**CCN2_TSS_Fwd**	CCCCAAACTCACACAACAACTC	AP-qPCR	This study
**CCN2_TSS_Rev**	GCGGCTGCCGTCGAG	AP-qPCR	This study
**TE1869_Fwd**	ATCAGCCAGTAGATAA	AP-qPCR	This study
**TE1869_Rev**	AGCGACTGGAGTAAGG	AP-qPCR	This study
**FOS_Fwd**	GGAGAATCCGAAGGGAAAGGA	qPCR	This study
**FOS_Rev**	GTTGGTCTGTCTCCGCTTGG	qPCR	This study
**CCN2_Fwd**	CGA GGA GTG GGT GTG TGA C	qPCR	This study
**CCN2_Rev**	TTC CAG TCG GTA AGC CGC	qPCR	This study
**KLF6_Fwd**	GAG GAG TAC TGG CAA CAG ACC T	qPCR	This study
**KLF6_Rev**	GCT GAA ACA TAG CAG GGC TC	qPCR	This study
**JUN_Fwd**	GAG CTG GAG CGC CTG ATA AT	qPCR	This study
**JUN_Rev**	CCC TCC TGC TCA TCT GTC AC	qPCR	This study

Table S3: List of siRNA sequences used in this study

Table S3: siRNA sequences used for transfection

siRNA	siRNA Sequence	References
**siSCR**	5'-AGGUAGUGUAAUCGCCUUG-3'	[1]
**siNuMA 1**	5'-GGUGGCAACUGAUGCUUUA-3'	[1]

References

1. Ray, S., Abugable, A.A., Parker, J., Liversidge, K., Palminha, N.M., Liao, C., Acosta-Martin, A.E., Souza, C.D.S., Jurga, M., Sudbery, I., et al. (2022). A mechanism for oxidative damage repair at gene regulatory elements. Nature *609*, 1038–1047. 10.1038/s41586-022-05217-8.

2. Hudson, J.J.R., Chiang, S.-C., Wells, O.S., Rookyard, C., and El-Khamisy, S.F. (2012). SUMO modification of the neuroprotective protein TDP1 facilitates chromosomal single-strand break repair. Nat Commun *3*, 733. 10.1038/ncomms1739.
