# Supplementary material for: ADP-ribosylation of NuMA promotes DNA single-strand break repair and transcription
Source: Cell Rep. 2025 May 20;44(6):115737. doi: 10.1016/j.celrep.2025.115737 (PMC12187637; doi:10.1016/j.celrep.2025.115737)
Supplement: Document S2. Article plus supplemental information [file mmc3.pdf]

Cell Reports

Article

ADP-ribosylation of NuMA promotes DNA single-strand break repair and transcription

Graphical abstract

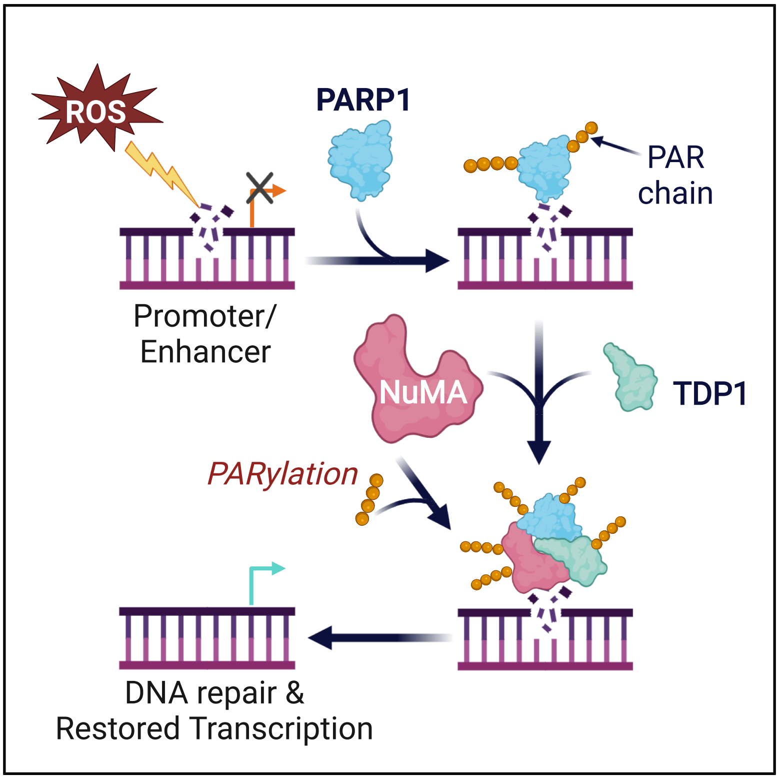

Highlights

- NuMA ADP-ribosylation at serine residues promotes DNA repair and transcription
- Mutating ADP-ribosylation sites in NuMA reduces its interaction with TDP1, affecting SSBR
- ADP-ribosylation of NuMA protects gene regulatory elements from oxidative breaks

Authors

Arwa A. Abugable, Chunyan Liao, Sarah Antar, Matthew Dowson, Sherif F. El-Khamisy

Correspondence

s.el-khamisy@bradford.ac.uk

In brief

Abugable et al. investigated the role of ADP-ribosylation in NuMA function during DNA repair. They demonstrate that ADP-ribosylation-deficient NuMA impairs repair kinetics and transcription and fails to protect selected gene regulatory elements from oxidative DNA breaks. This highlights the importance of ADP-ribosylation of NuMA in regulating transcription and DNA repair.

Abugable et al., 2025, Cell Reports *44*, 115737
June 24, 2025 © 2025 The Author(s). Published by Elsevier Inc.
https://doi.org/10.1016/j.celrep.2025.115737

CellPress

Cell Reports

CellPress
OPEN ACCESS

Article

ADP-ribosylation of NuMA promotes DNA single-strand break repair and transcription

Arwa A. Abugable,[1,2,5] Chunyan Liao,[1,2] Sarah Antar,[1,2,3] Matthew Dowson,[1,2] and Sherif F. El-Khamisy[1,2,4,6,*]
[1]School of Biosciences, Firth Court, University of Sheffield, Sheffield, UK
[2]The Healthy Lifespan and Neuroscience Institutes, University of Sheffield, Sheffield, UK
[3]Medical Biochemistry and Molecular Biology Department, Faculty of Medicine, Mansoura University, Mansoura, Egypt
[4]Institute of Cancer Therapeutics, School of Pharmacy and Medical Sciences, University of Bradford, Bradford, UK
[5]Present address: Department of Biochemistry, University of Oxford, South Parks Road, Oxford, UK
[6]Lead contact
*Correspondence: s.el-khamisy@bradford.ac.uk
https://doi.org/10.1016/j.celrep.2025.115737

SUMMARY

Single-strand breaks (SSBs) are prevalent DNA lesions implicated in genome instability. The nuclear mitotic apparatus protein (NuMA) has been reported to promote SSB repair (SSBR) and regulate transcription following oxidative stress. ADP-ribosylation, an important post-translational modification, regulates several processes, including chromatin remodeling, transcription, and DNA repair. To investigate its role in NuMA-dependent functions, we generated an ADP-ribosylation-deficient NuMA construct and report that NuMA ADP-ribosylation is required for its interaction with tyrosyl DNA phosphodiesterase 1 (TDP1), an SSBR player. Cells expressing ADP-ribosylation-deficient NuMA exhibit delayed SSBR kinetics following oxidative stress and reduced repair at promoter and enhancer regions, consistent with a role of NuMA in protecting non-coding regulatory regions from DNA damage. Furthermore, the expression of NuMA-regulated genes following oxidative stress requires ADP-ribosylation. Our findings demonstrate that ADP-ribosylation of NuMA promotes SSBR and transcription following oxidative stress, underscoring the importance of ADP-ribosylation in modulating DNA repair and gene expression.

INTRODUCTION

In mammalian cells, single-strand breaks (SSBs) represent the most prevalent DNA lesions, occurring approximately 10,000 times per cell daily.[1] Failure in their repair results in genome instability, disrupting vital cellular processes such as transcription and DNA replication.[2] SSBs and deficiencies in their repair have been implicated in various diseases, including cancer and neurological disorders.[3]

Oxidative stress is a major contributor to SSB formation, resulting in the direct disintegration of the oxidized bases/nucleotides or indirectly through the base excision repair pathway where SSB intermediates are formed.[4,5] These include abasic or apurinic/apyrimidinic sites (AP sites), which are formed after base loss or damage removal and undergo spontaneous β-elimination.[6] Programmed epigenetic processes, defined here as DNA and histone modifications that affect gene expression without changing the DNA sequence, can also lead to the formation of AP sites. For example, cytosine demethylation can generate reactive oxygen species (ROS), which oxidize guanine bases to 8-oxoguanine, which are then cleaved by 8-oxoguanine glycosylase (OGG1) to form AP sites.[7–10] This is in addition to histone demethylation by both flavin adenine dinucleotide-dependent monoamine oxidases and the Jumonji family, which generate ROS, further contributing to oxidative SSBs.[11–13]

SSB repair (SSBR) is a crucial repair pathway that maintains genomic integrity in cells through a series of precisely coordinated steps. Upon detection of an SSB, poly(ADP-ribose [ADPr]) polymerases (PARPs) recognize the lesion and catalyze the addition of ADPr chains to proteins and histones at the break sites, which facilitates the recruitment of repair proteins such as X-ray repair cross-complementing protein 1 (XRCC1).[14] XRCC1 interacts with and stabilizes the DNA end-processing enzymes such as tyrosyl DNA phosphodiesterase 1 (TDP1), aprataxin (APTX), apurinic/apyrimidinic endonuclease 1, and polynucleotide kinase 3′-phosphatase (PNKP).[15] SSBR then proceeds via the short-patch or long-patch repair pathway where the missing nucleotides are inserted, and the DNA strand is ligated.[16,17]

Recently, the nuclear mitotic apparatus protein (NuMA) has been implicated in SSBR.[18] NuMA helps protect gene regulatory elements such as promoters and enhancers from oxidative DNA damage. It facilitates the recruitment of SSBR components such as TDP1 to promoters, thereby contributing to an efficient repair process. The interaction between NuMA and TDP1 is mediated by ADP-ribosylation by PARP1. An increase in NuMA interaction with TDP1 is observed upon poly(ADPr) glycohydrolase (PARG) inhibitor treatment and *in vitro* in the presence of synthetic PAR. Furthermore, NuMA is implicated in the regulation of transcription of a specific cohort of NuMA-regulated genes (NRGs) following oxidative stress by promoting their expression.[18]

Check for updates

This is an open access article under the CC BY license (http://creativecommons.org/licenses/by/4.0/).

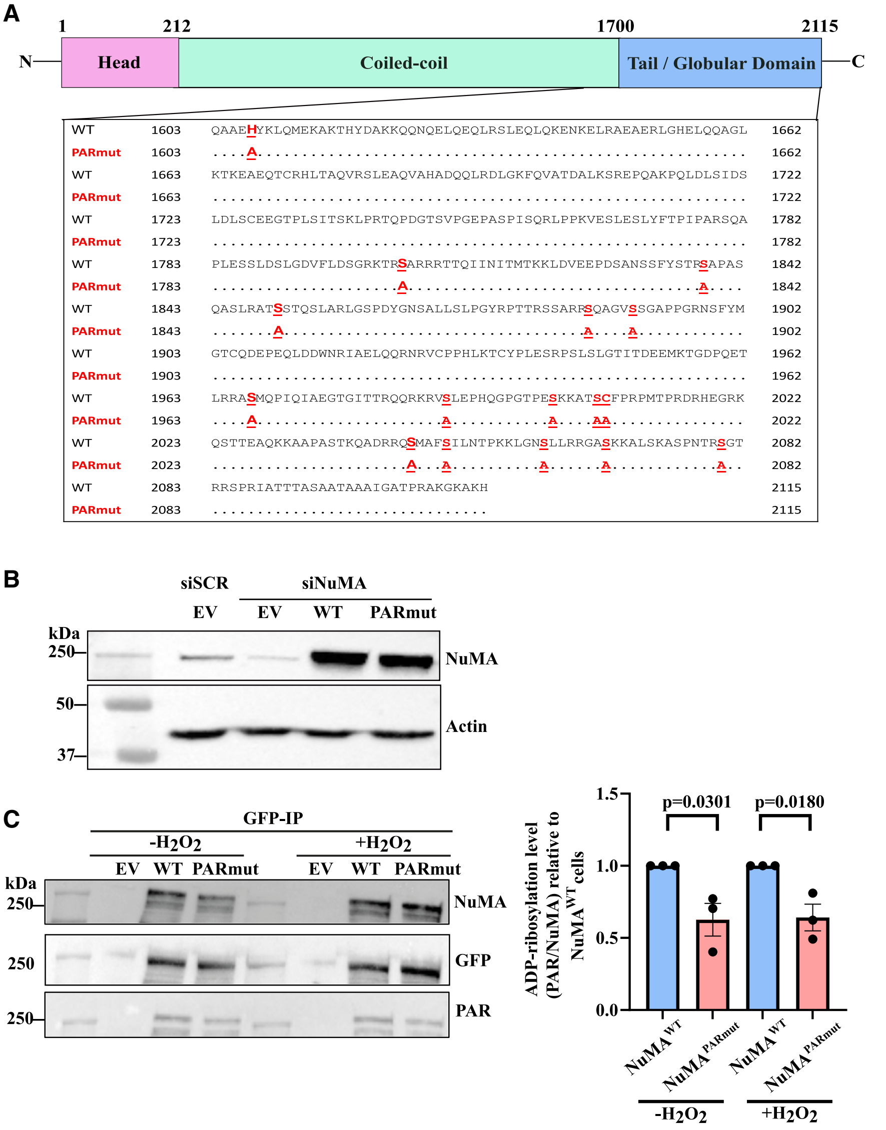

Figure 1. Generation and validation of ADP-ribosylation-deficient construct

(A) Schematic illustration highlighting the head, coiled-coil, and tail (C terminus globular domain) of NuMA. The sites susceptible to ADP-ribosylation are shown and highlighted in bold and red for NuMA^{WT}, and the subsequent mutated residue, alanine, is highlighted in red for NuMA^{PARmut}.

(B) Representative immunoblotting from whole-cell extracts showing the successful knockdown of endogenous NuMA with siRNA and transfection of the empty vector (EV), NuMA^{WT}, and NuMA^{PARmut} constructs.

(C) Representative immunoblotting following immunoprecipitation with GFP-trap beads of GFP-NuMA^{WT} and GFP-NuMA^{PARmut} in cells that were either untreated or treated with 10 μM H_2O_2 for 10 min on ice in the dark. Bar chart shows the fold change in relative ADP-ribosylation levels (PAR/NuMA) in NuMA^{PARmut} relative to NuMA^{WT}. The bar chart represents data collected from three biological replicates, with error bars representing the standard error of the mean. Two-sided unpaired Student's t test was conducted.

NRGs are genes that are upregulated in NuMA-proficient cells but downregulated in NuMA-deficient cells following oxidative stress. The regulation of the expression of NRGs is achieved by facilitating the release of paused RNA polymerase II (RNAPII) molecules from the promoter proximal regions through limiting their ADP-ribosylation in the presence of NuMA.[18]

ADP-ribosylation is a reversible post-translational modification (PTM) crucial for various cellular processes. It involves the transfer of the ADPr moiety from NAD^+ to nucleic acids or different amino acid side chains in proteins. NuMA is indirectly ADP-ribosylated by tankyrase 1 in a DNA-independent manner, which impacts its function in stabilizing mitotic spindles and telomeres.[19] It also interacts with PARP3, which ADP-ribosylates NuMA directly in a DNA-dependent manner, and this is important in DNA double-strand break (DSB) repair[20] and mitosis.[21] During the DNA damage response (DDR), the ADPr chains are synthesized by PARPs such as PARP1 and are removed by PARG and ADP-ribosyl hydrolase 3 (ARH3). Early insights into the amino acid selectivity of PARP1 revealed that lysine,[22,23] arginine,[24] aspartate, and glutamate[25] are the primary target residues for ADP-ribosylation. However, more recent mass spectrometry studies have revealed that serine residues in several proteins and histones are the primary sites that are susceptible to ADP-ribosylation in response to DNA damage.[26–28]

Following oxidative damage, NuMA was reported to be susceptible to ADP-ribosylation at 16 residues.[29] The C terminus globular domain, which is sufficient and indispensable for SSBR, harbors 15 out of these 16 residues.[18] Notably, 14 out of these 15 residues are serine, which is the major amino acid residue susceptible to ADP-ribosylation following DNA damage.[27] Here, we mutated these residues to generate ADP-ribosylation-deficient NuMA (NuMA^{PARmut}), which revealed that NuMA ADP-ribosylation is important for the timely repair of SSBs and for promoting the transcription of NRGs following oxidative stress.

RESULTS

Generation of NuMA^{PARmut}

The serine ADP-ribosylation sites in NuMA that were identified by mass spectrometry[29] were mutated in GFP-tagged NuMA constructs (NuMA^{WT}) to alanine to generate the NuMA^{PARmut} construct (Figure 1A). The position of the 16 ADP-ribosylation sites and the amino acid sequence in the NuMA^{WT} and NuMA^{PARmut} constructs are described in Table S1. Both constructs were resistant to small interfering RNA (siRNA) targeting endogenous NuMA. This was

CellPress
OPEN ACCESS

A

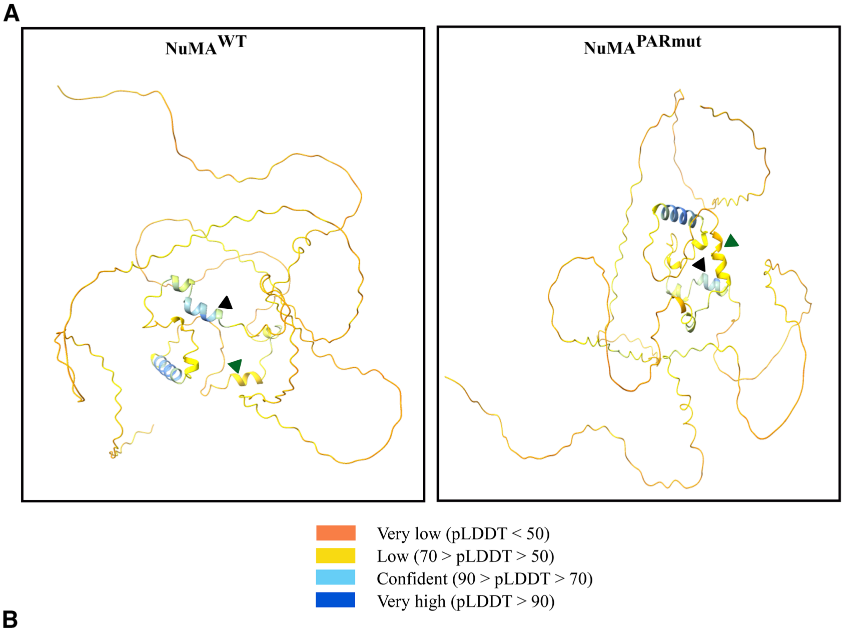

B

NuMA^{WT}

QVSILNRQVLEKEGESKELKRLVMAESEKSQKLEERLRLLQAETASNSARAAERSSALREEVQSLREEAEKQRVASENLRQ
ELTSQAERAEELGQELKAWQEKFFQKEQALSTLQLEHTSTQALVSELLPAKHLCQQLQAEQAAAEKRHREELEQSKQAAGG
LRAELLRAQRELGELIPLRQKVAEQERTAQQLRAEKASYAEQLSMLKKAHGLLAEENRGLGERANLGRQFLEVELDQAREK
YVQELAAVRADAETRLAEVQREAQSTARELEVMTAKYEGAKVKVLEERQRFQEERQKLTAQVEQLEVFQREQTKQVEELSK
KLADSDQASKVQQQKLKAVQAQGGESQQEAQRLQAQLNELQAQLSQKEQAAEHYKLQMEKAKTHYDAKKQQNQELQEQLRS
LEQLQKENKELRAEAERLGHELQQAGLKTKEAEQTCRHLTAQVRSLEAQVAHADQQLRDLGKFQVATDALKSREPQAKPQL
DLSIDSLDLSCEEGTPLSITSKLPRTQPDGTSVPGEPASPISQRLPPKVESLESLYFTPIPARSQAPLESSLDSLGDVFLD
SGRKTRSARRRTTQIINITMTKKLDVEEPDSANSSFYSTRSAPASQASLRATSSTQSLARLGSPDYGNSALLSLPGYRPTT
RSSARRSQAGVSSGAPPGRNSFYMGTCQDEPEQLDDWNRIAELQQRNRVCPPHLKTCYPLESRPSLSLGTITDEEMKTGDP
QETLRRASMQPIQIAEGTGITTRQQRKRVSLEPHQGPGTPESKKATSCFPRPMTPRDRHEGRKQSTTEAQKKAAPASTKQA
DRRQSMAFSILNTPKKLGNSLLRRGASKKALSKASPNTRSGTRRSPRIATTTASAATAAAIGATPRAKGKAKH

NuMA^{PARmut}

QVSILNRQVLEKEGESKELKRLVMAESEKSQKLEERLRLLQAETASNSARAAERSSALREEVQSLREEAEKQRVASENLRQ
ELTSQAERAEELGQELKAWQEKFFQKEQALSTLQLEHTSTQALVSELLPAKHLCQQLQAEQAAAEKRHREELEQSKQAAGG
LRAELLRAQRELGELIPLRQKVAEQERTAQQLRAEKASYAEQLSMLKKAHGLLAEENRGLGERANLGRQFLEVELDQAREK
YVQELAAVRADAETRLAEVQREAQSTARELEVMTAKYEGAKVKVLEERQRFQEERQKLTAQVEQLEVFQREQTKQVEELSK
KLADSDQASKVQQQKLKAVQAQGGESQQEAQRLQAQLNELQAQLSQKEQAAEAYKLQMEKAKTHYDAKKQQNQELQEQLRS
LEQLQKENKELRAEAERLGHELQQAGLKTKEAEQTCRHLTAQVRSLEAQVAHADQQLRDLGKFQVATDALKSREPQAKPQL
DLSIDSLDLSCEEGTPLSITSKLPRTQPDGTSVPGEPASPISQRLPPKVESLESLYFTPIPARSQAPLESSLDSLGDVFLD
SGRKTRAARRRTTQIINITMTKKLDVEEPDSANSSFYSTRAAPASQASLRATASTQSLARLGSPDYGNSALLSLPGYRPTT
RSSARRAQAGVASGAPPGRNSFYMGTCQDEPEQLDDWNRIAELQQRNRVCPPHLKTCYPLESRPSLSLGTITDEEMKTGDP
QETLRRAAMQPIQIAEGTGITTRQQRKRVALEPHQGPGTPEAKKATAAFPRPMTPRDRHEGRKQSTTEAQKKAAPASTKQA
DRRQAMAFAILNTPKKLGNALLRRGAAKKALSKASPNTRAGTRRSPRIATTTASAATAAAIGATPRAKGKAKH

Figure 2. NuMA^{WT} and NuMA^{PARmut} exhibit highly disordered structures

(A) Predicted structure of NuMA^{WT} and NuMA^{PARmut} as generated by AlphaFold2. The colors used correspond to AlphaFold2's predicted local distance difference test (pLDDT) values of the AlphaFold2 structures. The green and black triangles represent the positions of the serine and corresponding alanine residues in NuMA^{WT} and NuMA^{PARmut} that lie within helices.

(B) The amino acid sequences of NuMA^{WT} and NuMA^{PARmut}. The ADP-ribosylation sites are shown in red, bold, and underlined. The amino acids corresponding to the helical structures are highlighted in yellow.

important so that after the depletion of endogenous NuMA, overexpression of the GFP-tagged ADP-rebosylation-proficient NuMA (NuMA^{WT}) and NuMA^{PARmut} constructs could be conducted, followed by immunoblotting, to rule out that any observed effect is due to the presence of endogenous NuMA (Figure 1B). Immunoprecipitation of the GFP-tagged NuMA^{WT} and NuMA^{PARmut} using GFP-trap beads was conducted under both unperturbed conditions and following treatment with H_2O_2. This was followed by anti-PAR immunoblotting, which revealed that NuMA^{PARmut} had an approximately 40% decrease in the levels of ADP-ribosylation under both untreated and H_2O_2-treated conditions compared to NuMA^{WT} (Figure 1C). These results demonstrate that NuMA^{PARmut} exhibits reduced ADP-ribosylation levels compared to NuMA^{WT}.

NuMA^{PARmut} exhibits structure similar to that of NuMA^{WT}

To investigate whether any anticipated phenotypes observed in NuMA^{PARmut} are a consequence of the ADP-ribosylation deficiency or due to induced structural differences, the structures of NuMA^{WT} and NuMA^{PARmut} were examined using AlphaFold.[30,31] Modeling of the C terminus globular domain using only AlphaFold2 suggests that it is a highly disordered structure, with several short regions of helical structure predicted toward the center (Figure 2A). Disordered regions tend to have a higher evolutionary rate, suggesting that these regions are more resistant to amino acid substitutions.[32] However, 2 of the 16 ADP-ribosylation sites that have been mutated in NuMA^{PARmut} do lie within potentially structured regions (Figures 2A and 2B). The predicted structure from AlphaFold2 suggests that as a result of mutating the serine residue at position 1887, there could be a helix composed of 10 amino acids in NuMA^{PARmut} compared to 7 amino acids in NuMA^{WT}. However, mutation of the serine residue at position 1969 results in no difference in the length of the helix between both proteins (Figure 2B). Although the remaining helices in both proteins remain of similar length and occur at similar positions, it is also worth noting that there are two short 3-amino acid helices in NuMA^{PARmut} covering the positions 1863–1865 and 1870–1872. There is only one short 3-amino acid helix in NuMA^{WT} covering the positions 1868–1871. None of these helices include ADP-ribosylation sites that have been mutated (Figure 2B).

Modeling of the full-length NuMA^{WT} and NuMA^{PARmut} using AlphaFold3 also revealed a disordered structure of the C terminus globular domain (Figure S1A). However, when modeled in complex with TDP1, the C terminus globular domain in NuMA^{WT} and NuMA^{PARmut} appears to undergo a structural change whereby it forms a globular domain that interacts with TDP1 (Figure S1B). Given that NuMA has been reported to perform its mitotic role as a dimer[33] and that there is no evidence to date about the stoichiometry of NuMA required for its oxidative DNA break repair role, modeling of NuMA^{WT} and

NuMA^{PARmut} as a dimer was also conducted. This revealed that the C terminus was also structured into a globular domain for both NuMA^{WT} and NuMA^{PARmut} (Figure S1C) and that TDP1 was found to interact with their C terminus globular domain (Figure S1D). Furthermore, we have also used AlphaFold3 to model the structure of NuMA^{WT} and NuMA^{PARmut} in both their monomer and dimer conformations when bound to DNA, which corresponded to the sequence of the *FOS* promoter region. The structures obtained were quite similar, with no significant differences observed between the two proteins (Figures S1E and S1F). It is worth noting that although these structures show low prediction confidence scores, including the interface predicted template modeling and the predicted local distance difference test (pLDDT) scores (Figure 2A), upon modeling with AlphaFold2 and AlphaFold3, there are no major structural differences observed between both proteins. These findings suggest that the amino acid modifications introduced in NuMA^{PARmut} are predicted not to change its structure compared to NuMA^{WT} and that any findings reported as a result of this mutation could be attributed to the ADP-ribosylation deficiency.

Decreased interaction of TDP1 with NuMA^{PARmut}

We previously reported that the interaction between NuMA and TDP1 is ADP-ribosylation dependent.[18] To examine whether the ADP-ribosylation of NuMA itself promotes this interaction, we compared NuMA^{WT} and NuMA^{PARmut} immunocomplexes for the presence of TDP1, PARP1, and XRCC1. Immunoprecipitation using GFP-trap beads was conducted on lysates obtained from cells transfected with either NuMA^{WT} or NuMA^{PARmut} and either untreated or treated with H_2O_2. In both the untreated and H_2O_2-treated cells, there was no difference observed in the interaction between PARP1 and XRCC1 with either NuMA^{WT} or NuMA^{PARmut} (Figure 3A). However, 38% and 45% decreases in the interaction between TDP1 and NuMA^{PARmut} were observed in both untreated and H_2O_2-treated cells, respectively (Figure 3A). This suggests that ADP-ribosylation of NuMA promotes its interaction with TDP1, but not with PARP1 and XRCC1. This was then further investigated in the chromatin fraction, where similar results were observed in H_2O_2-treated cells. The interaction between TDP1 and NuMA^{PARmut} was 50% lower than that between TDP1 and NuMA^{WT} (Figure 3B). These results suggest that ADP-ribosylation of NuMA at the 14 serine amino acids in the C terminus globular domain mediates the interaction with TDP1.

NuMA^{PARmut} is defective in SSBR

To test whether the role of NuMA in SSBR is dependent on the ADP-ribosylation of its serine residues, MRC5 cells were treated with H_2O_2 and subjected to alkaline comet assays to investigate the repair kinetics over 30 and 60 min in H_2O_2-free media. It is worth noting that the alkaline comet assay measures both SSBs and DSBs, but H_2O_2 predominantly induces SSBs, hence, this assay primarily measures SSBR kinetics. As previously reported,[18] NuMA-deficient cells had longer comet tails, suggestive of an increased level of DNA breakage, when compared to cells transfected with scrambled non-targeting siRNA (Figure 4A). Complementation of the NuMA-deficient cells with the NuMA^{WT} restored the repair defect observed in NuMA-deficient cells. However, complementation with the NuMA^{PARmut} did not (Figure 4A). Analysis of the percentage of DNA breaks remaining following recovery in H_2O_2-free media revealed that after 30 min, NuMA-deficient cells possessed 44% DNA breaks compared to 36% in the NuMA-proficient cells. Complementation of the NuMA-deficient cells with NuMA^{WT} decreased the percentage of remaining DNA breaks to 30%, whereas complementation with NuMA^{PARmut} decreased it to only 50% (Figure 4B). Following a 60-min recovery, NuMA-deficient cells contained 33% of breaks compared to only approximately 18% in the NuMA-proficient cells and NuMA-deficient cells complemented with NuMA^{WT}. However, complementation with NuMA^{PARmut} led to 35% DNA breaks remaining, which is similar to the NuMA-deficient cells (Figure 4B). These findings indicate that serine ADP-ribosylation deficiency of NuMA leads to increased DNA damage levels and delay in the repair of the oxidative DNA breaks.

Following generation of NuMA^{PARmut} and characterizing it, a mutation was identified in the NuMA^{WT} construct, which was used subsequently to generate NuMA^{PARmut}. As a result of this mutation, at position 1528, which is in the coiled-coil domain, a lysine residue was present instead of glutamic acid. This was corrected to generate the NuMA^{Corrected} construct, which was successfully expressed (Figure S2A). To investigate whether this mutation had an impact on the role of NuMA in mediating SSBR, an alkaline comet assay was conducted. Complementation of the NuMA-deficient cells with the NuMA^{WT} and NuMA^{Corrected} restored the repair defect observed in NuMA-deficient cells to similar levels (Figure S2B). Analysis of the percentage of DNA breaks remaining following recovery in H_2O_2-free media revealed that after 30 min, NuMA-deficient cells possessed 52% DNA breaks compared to 34% in the NuMA-proficient cells. Complementation of the NuMA-deficient cells with NuMA^{WT} and NuMA^{Corrected} decreased the percentage of remaining DNA breaks to 38% for both conditions (Figure S2C). Following a 60-min recovery, NuMA-deficient cells had reduced the percentage of DNA breaks remaining to 43%, while the NuMA-proficient cells were at 20%. Complementation of the NuMA-deficient cells with NuMA^{WT} and NuMA^{Corrected} resulted in 26% and 23% remaining DNA breaks, respectively, which is similar to the NuMA-proficient cells. However, complementation with NuMA^{PARmut} led to 35% DNA breaks remaining, which is similar to the NuMA-deficient cells (Figure S2C). These findings indicate that the missense mutation E1528K in the NuMA^{WT} construct did not affect the role of NuMA in SSBR.

NuMA^{PARmut} fails to protect gene regulatory elements from oxidative DNA breaks

Given that NuMA protects promoters from oxidative DNA breaks,[18] the level of oxidative damage at the promoters of selected NRGs was assessed using OGG1-AP-qPCR. AP-qPCR allows assessment of the level of AP sites, and in combination with *in vitro* digestion with OGG1, it enables the capture of 8-oxoguanine residues that have not been excised *in vivo*. NuMA-deficient cells were found to possess 1.5-, 1.9-, and 2.9-fold increases in the levels of AP sites at the promoters of *FOS*, *CCN2*, and *SRF*, respectively, relative to NuMA-proficient cells, which is in line with previously reported findings that

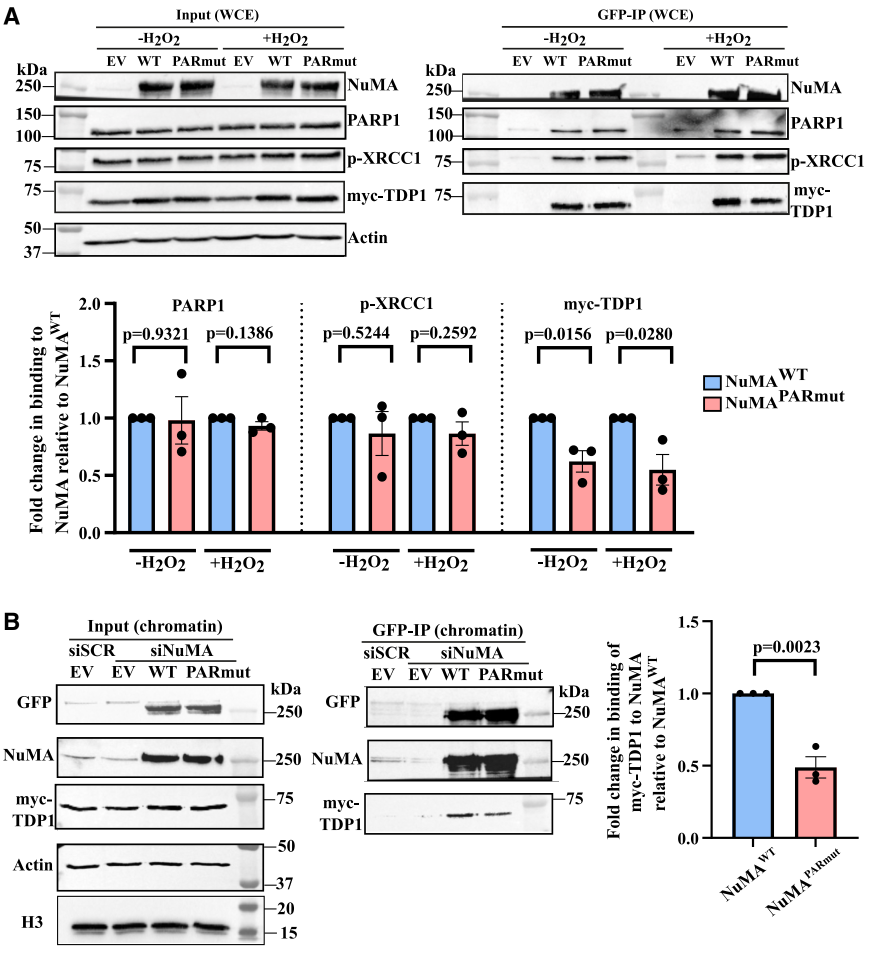

Figure 3. ADP-ribosylation-deficient NuMA shows decreased TDP1 interaction

(A) Representative immunoblotting from immunoprecipitation with GFP-trap beads of the GFP-NuMA^{WT} and GFP-NuMA^{PARmut} in whole-cell extracts of untreated and H_2O_2-treated (10 μM H_2O_2 for 10 min on ice in the dark) cells that were also co-transfected with myc-TDP1. Actin was used as a loading control. Bar charts show the fold change in binding of PARP1, XRCC1, and myc-TDP1 to NuMA in NuMA^{PARmut} relative to NuMA^{WT}. The bar chart represents data from three biological replicates, with error bars representing the standard error of the mean. Two-sided unpaired Student's t test was conducted.

(B) Representative immunoblotting from immunoprecipitation with GFP-trap beads of the GFP-NuMA^{WT} and GFP-NuMA^{PARmut} in the chromatin fraction of cells co-transfected with myc-TDP1 and treated with 10 μM H_2O_2 for 10 min on ice in the dark. H3 was used as a loading control. Bar chart shows the fold change in binding of myc-TDP1 to NuMA in NuMA^{PARmut} relative to NuMA^{WT}. The bar chart represents data collected from three biological replicates with error bars representing the standard error of the mean. Two-sided unpaired Student's t test was conducted.

promoters of NuMA-deficient cells possess more oxidative breaks[18] (Figure 4C). It is also consistent with the presence of DNA repair hotspots at promoters.[34,35] The complementation of the NuMA-deficient cells with NuMA^{WT} restored the levels of AP sites close to those of the WT (non-transfected cells). This was in contrast to complementation with the NuMA^{PARmut}, resulting in 1.5-, 2.4-, and 2.6-fold increases in the levels of AP sites relative to the control non-transfected cells, which was similar to that observed in the NuMA-deficient cells (Figure 4C).

Given that NuMA also plays a role in protecting enhancers from oxidative DNA damage,[18] OGG1-AP-qPCR was conducted to assess the level of oxidative breaks at the *TE6189* enhancer, which has been reported to regulate two of the NRGs, *ATF7JP* and *ZCRB1*.[36] NuMA-deficient cells were found to possess a 1.27-fold increase in the levels of AP sites compared to the control non-transfected cells (Figure 4D). In contrast, complementing the NuMA-deficient cells with NuMA^{WT} restored the level of AP sites to levels similar to those of the non-transfected cells, whereas complementation with NuMA^{PARmut} resulted in a 2-fold increase in the levels of AP sites (Figure 4D). This is also in line with reports suggesting that enhancers are hotspots of DNA damage.[34,37]

Since AP sites were enriched at promoters and enhancers known to be protected by NuMA, the enrichment of NuMA^{WT} and NuMA^{PARmut} was investigated using chromatin immunoprecipitation (ChIP)-qPCR at the *FOS* promoter and *TE6189* enhancer. The recruitment of NuMA^{PARmut} to the *FOS* promoter and *TE6189* enhancer was found to be impaired compared to NuMA^{WT}, which suggests that NuMA ADP-ribosylation is required for its recruitment to the SSB sites in response to oxidative stress (Figure S3). These findings demonstrate that serine ADP-ribosylation of NuMA is required for its role to protect promoters and enhancers from oxidative DNA damage.

NuMA^{PARmut} fails to rescue transcription defects

NuMA is also known to play a role in promoting the transcription of a certain cohort of NRGs, which includes immediate-early response genes (IERGs), pro-inflammatory genes, and paused genes.[18] To investigate whether ADP-ribosylation of NuMA is required for this role, qPCR of some of these NuMA-regulated, paused IERGs (*FOS*, *CCN2*, *JUN*, and *KLF6*) was conducted. The expression level of these genes following a 90-min recovery in H_2O_2-free media was assessed relative to the expression of these genes in the scrambled-transfected untreated cells as previously described.[18] NuMA depletion resulted in 77%, 40%, 41%, and 53% decreases in the expression of *FOS*, *JUN*, *CCN2*, and *KLF6*, respectively, following oxidative stress, in line with our previous findings[18] (Figures 5 and S4). Complementation of NuMA-deficient cells with NuMA^{WT} increased the

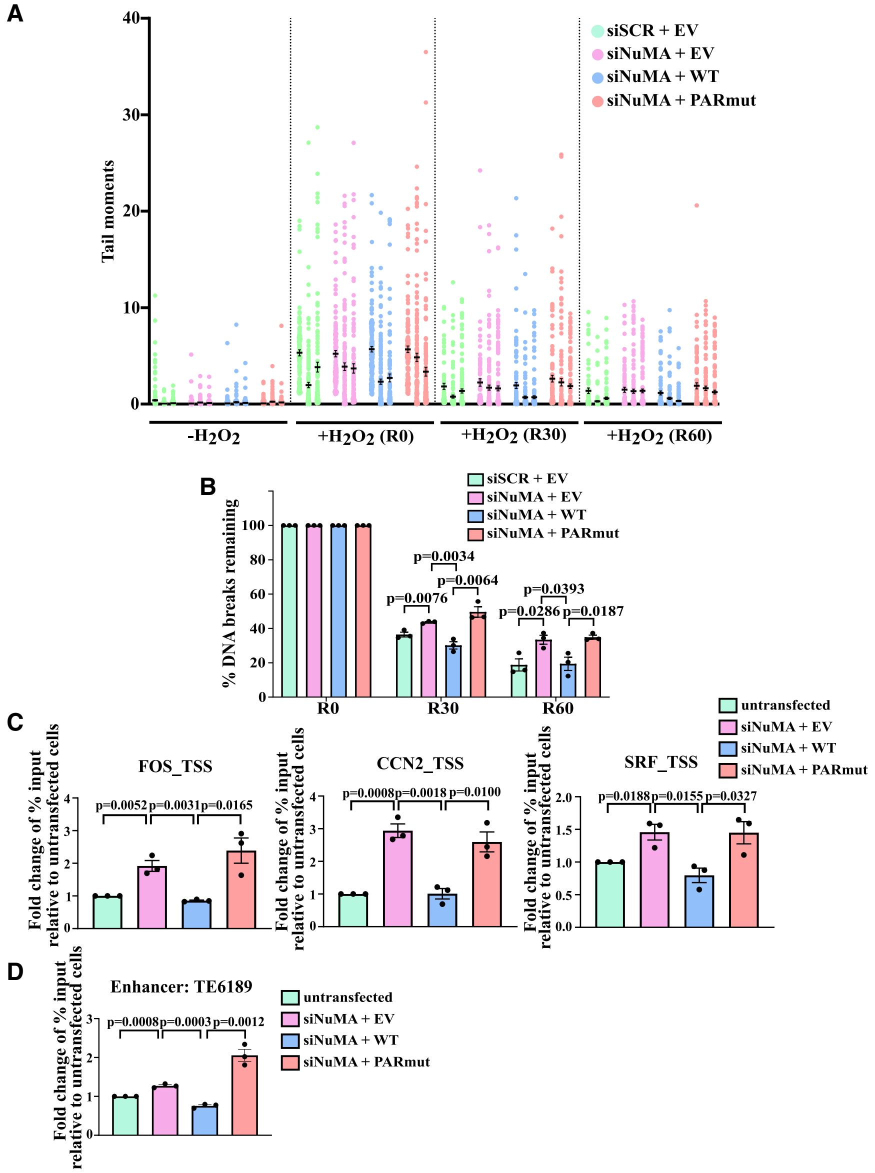

Figure 4. ADP-ribosylation-deficient NuMA demonstrates delayed SSBR kinetics and increased AP sites at promoters and enhancers

Cells were transfected with siSCR or siNuMA and then complemented with either EV, NuMA^{WT}, or NuMA^{PARmut} constructs. The cells were left untreated or treated with 20 μM H_2O_2 for 10 min on ice in the dark, followed by recovery in H_2O_2-free media for 0, 30, and 60 min, denoted as R0, R30, and R60, respectively, before being subjected to alkaline comet assay.

(A) Violin plot showing the distribution of the comet tail moments at the indicated time points. The data shown are from three biological replicates.

(B) Bar plot showing the percentage of DNA breaks remaining during the recovery time points. The bar chart represents data from three biological replicates, with error bars representing the standard error of the mean. Two-sided unpaired Student's t test was conducted.

(C and D) Cells were transfected with siSCR or siNuMA and then complemented with either EV, NuMA^{WT}, or NuMA^{PARmut} constructs. OGG1-AP-qPCR was conducted at (C) promoters of *FOS*, *CCN2*, and *SRF* and (D) the *TE6189* enhancer. Bar charts show the fold change in percentage of input relative to the untransfected cells. The bar chart represents data from three biological replicates, with error bars representing the standard error of the mean. Two-sided unpaired Student's t test was conducted.

expression of *FOS*, *JUN*, *CCN2*, and *KLF6* to levels similar to those observed with the scrambled-transfected cells, suggesting a rescue in the transcription defect observed in the NuMA-deficient cells. However, complementation with NuMA^{PARmut} failed to rescue the observed defect in expression, with the expression of *FOS*, *JUN*, *CCN2*, and *KLF6* still found to be decreased by 28%, 29%, 32%, and 25%, respectively, relative to NuMA^{WT} (Figures 5 and S4). This level of reduction in expression is similar to the levels observed in NuMA-deficient cells, suggesting that transcription recovery is defective when NuMA is serine ADP-ribosylation deficient. It is worth noting that cells complemented with NuMA^{PARmut} also exhibited reduced transcription levels of *FOS*, *JUN*, *CCN2*, and *KLF6* compared to the NuMA^{WT} cells following 48 h of serum starvation and 90-min recovery in serum-containing media, without H_2O_2 treatment (Figures 5 and S4). While this difference was more pronounced in H_2O_2-treated cells, a similar trend was observed in the untreated cells, albeit not statistically significant, which suggests that the damage could be caused by endogenous sources of oxidative stress. These results highlight that serine ADP-ribosylation of NuMA promotes its role in transcription independently of the source of oxidative stress.

To confirm whether the change in incubation temperature during the treatment protocol (37°C during serum starvation, ice for H_2O_2 treatment, and 37°C for the recovery in serum-containing media) affects the observed transcriptional response, we measured the transcript levels following incubation on ice. We specifically assessed the transcript levels of *JUN*, *CCN2*, and *KLF6* after incubation on ice in the absence and presence of H_2O_2. Our results show that the cold shock does not affect the expression of the NRGs, for both untreated and H_2O_2-treated cells (Figure S5). These data indicate that the observed transcriptional response to recovery in serum-containing media is not affected by the preceding change in temperature.

Given that NuMA acts as a "PAR sink" and limits the ADP-ribosylation of RNAPII, increasing its availability at the promoters,[18] we investigated whether NuMA^{PARmut} would affect the enrichment of RNAPII at the promoters. Using the Pan RNAPII antibodies, ChIP-qPCR at the *CCN2* promoter revealed

CellPress
OPEN ACCESS

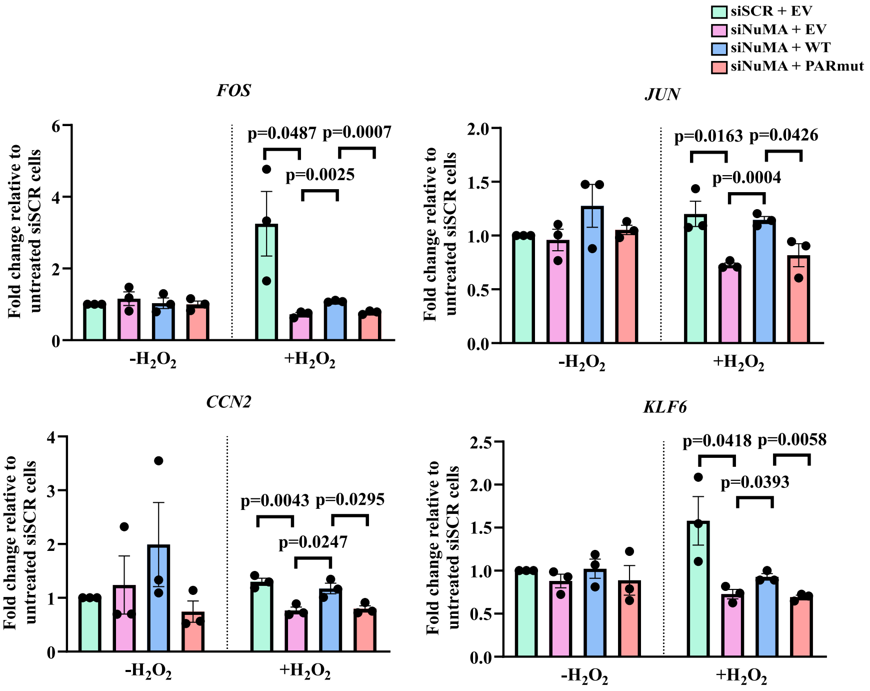

Figure 5. ADP-ribosylation-deficient NuMA leads to decreased expression of NuMA-regulated genes

Cells were transfected with siSCR or siNuMA and then complemented with either EV, NuMA^{WT}, or NuMA^{PARmut} constructs. They were serum starved for 48 h and left either untreated (-H_2O_2) or treated with 10 μM H_2O_2 (+H_2O_2) for 10 min on ice in the dark and recovered in serum-containing media for 90 min. qPCR measuring the expression of transcripts was conducted and represented as a fold change, where untreated and siSCR H_2O_2-treated cells were normalized to siSCR-untreated cells, while all other conditions were normalized to siSCR H_2O_2-treated cells. The bar chart represents data from three biological replicates, with error bars representing the standard error of the mean. Two-sided unpaired Student's t test was conducted.

that in the NuMA^{PARmut}-complemented cells, a 50% decrease in the enrichment of RNAPII was observed, which was similar to that observed in the NuMA-deficient cells compared to the siSCR+EV (empty vector) or the NuMA^{WT}-complemented cells (Figure S6). This suggests that serine ADP-ribosylation of NuMA is important for facilitating the recruitment of RNAPII at the promoters in response to oxidative stress.

TDP1 overexpression does not rescue NuMA^{PARmut}-SSBR and transcription defects

To investigate whether TDP1 overexpression could rescue the repair defects associated with NuMA deficiency or ADP-ribosylation deficiency, we examined the SSBR kinetics in NuMA-deficient and NuMA^{PARmut}-complemented cells using the alkaline comet assay. TDP1 overexpression did not improve the repair kinetics in either condition (Figures S7A and S7B). Since the global increase in TDP1 levels resulting from the overexpression may not necessarily occur at the SSB sites affected by NuMA deficiency or ADP-ribosylation deficiency, OGG1-AP-qPCR at specific genomic loci, such as the *FOS*, *CCN2*, and *SRF* promoters and *TE6189* enhancer, was conducted. This revealed a reduction in the AP site accumulation upon TDP1 overexpression in both NuMA-deficient and NuMA^{PARmut}-complemented cells; however, this reduction was statistically significant only in the NuMA-deficient cells (Figures S7C and S7D). This suggests that while TDP1 overexpression may partially compensate for the loss of NuMA, it is insufficient to fully restore the repair defect associated with the ADP-ribosylation deficiency of NuMA.

We further examined the impact of TDP1 overexpression on transcriptional regulation by assessing the expression of *FOS*, *CCN2*, *JUN*, and *SRF*. Interestingly, TDP1 overexpression successfully rescued the transcriptional defects in the NuMA-deficient cells but failed to do so in the NuMA^{PARmut}-complemented cells (Figure S7E). This finding reinforces our model that NuMA ADP-ribosylation plays an important role in facilitating the transcriptional response following oxidative stress beyond its key role in SSBR.

DISCUSSION

This study elucidates the critical role of ADP-ribosylation in mediating the role of NuMA in SSBR and transcription regulation following oxidative stress. By generating the NuMA^{PARmut} construct, which lacks ADP-ribosylation sites in the C terminus globular domain that is known to play a role in mediating SSBR, we provide evidence for the importance of NuMA ADP-ribosylation in facilitating its interaction with TDP1 and promoting SSBR and transcription following oxidative damage. This highlights the intricate regulatory mechanisms governing the DNA repair processes in cells and underscores the essential role of NuMA ADP-ribosylation in ensuring the efficient repair of oxidative DNA breaks.

The investigation into the generation of ADP-ribosylation-deficient proteins and their consequences has not been extensively explored. To our knowledge, NuMA^{PARmut} is the first ADP-ribosylation-deficient heteromodified DNA repair protein. There are three reports to date on ADP-ribosylation-deficient proteins; of these, PARP1 is a DNA repair protein that is automodified.[38] This is in addition to androgen receptor (AR) in mammalian cells[39] and H2B in *Dictyostelium*.[40] The ADP-ribosylation sites in PARP1 and AR were also identified through mass spectrometry studies. In PARP1, 3 serine residues were mutated to alanine,[38] whereas in AR, 11 cysteine residues were mutated to glycine.[39] The ADP-ribosylation-deficient PARP1 demonstrated decreased PARP1 automodification and enhanced PARP1 trapping, whereas ADP-ribosylation-deficient AR was found to abolish binding to PARP9. The generation of

NuMA^{PARmut} did not completely abolish the ADP-ribosylation of NuMA and resulted in a residual 60% ADP-ribosylation level compared to NuMA^{WT} and therefore did not fully eliminate binding to TDP1. This suggests the possible presence of additional ADP-ribosylation sites not identified by the mass spectrometry study; these therefore were not mutated in NuMA^{PARmut}. This aligns with the report demonstrating how the mutation of just the three serine residues in PARP1 decreased but did not abolish PARP1 ADP-ribosylation levels.[38] Additionally, mutation of 4 out of the 11 ADP-ribosylation sites of AR resulted in a reduction, rather than complete abolishment, in the ADP-ribosylation levels and PARP9 binding. It is also in line with the report on the mutation of a single glutamic acid residue to alanine in H2Bv3 (E18) in *Dictyostelium*, which resulted in a slight reduction in ADP-ribosylation levels.[40] In contrast, mutating E19 in H2Bv3 to alanine had no impact on ADP-ribosylation. However, simultaneous mutation of both E18 and E19 resulted in complete loss of the ADP-ribosylation signal.

As is evident from the partial decrease, rather than a complete abolishment of the ADPr signal observed in the NuMA^{PARmut}, it is likely that not all ADP-ribosylation sites in NuMA have been mutated. These residual ADP-ribosylation sites may still facilitate interactions with the remaining pool of TDP1, resulting in the partial recruitment of TDP1 to the chromatin. Furthermore, TDP1 itself is susceptible to various PTMs, including ADP-ribosylation at S172, S180, and H130 by PARP1,[29] which were not disrupted in our experimental settings. These ADP-ribosylated residues in TDP1 could have facilitated the interaction of TDP1 with NuMA and its subsequent recruitment to the chromatin. This hypothesis aligns with previous reports on various PTMs that regulate the recruitment of TDP1 to topoisomerase 1 cleavage complexes (TOP1ccs), the primary lesion repaired by TDP1. These PTMs include ADP-ribosylation by PARP1,[41] phosphorylation at S81 by ATM and DNA-PK,[42,43] SUMOylation at K111 by Ubc9,[44] arginine methylation by PRMT5 at R361 and R586,[45] and deubiquitination by UCHL3.[46] All of these modifications stabilize TDP1 and enhance its recruitment to TOP1cc break sites.[47,48] These findings support the idea that multiple PTMs regulate the stability of TDP1 and function, opening avenues for investigating how different PTMs might regulate the role of NuMA in oxidative DNA break repair and transcription regulation.

Utilizing AlphaFold to predict the structure of the C terminus globular domain of both NuMA^{WT} and NuMA^{PARmut}, which are known for their importance in mediating the SSBR role of NuMA and contain the 16 ADP-ribosylation sites in NuMA, revealed a highly disordered structure with a few helices in the center, which had minor alterations in the length of the amino acids constituting the helices. Although histidine-to-alanine substitutions in disordered proteins have been reported to affect the biophysical properties of certain proteins,[49] the mutations introduced in NuMA^{PARmut} are predicted not to impact the structure or flexibility of the rest of the disordered region as previously reported.[50] It is also worth acknowledging the limitations of AlphaFold2 in predicting the structure of intrinsically disordered proteins, especially that the pLDDT values, which are a per-residue model confidence score, are less than 50 for most of the structures. Moreover, as a tool, AlphaFold has not been validated thoroughly yet for predicting the consequences on protein structure following amino acid substitutions.

ADP-ribosylation of the serine residues has been implicated in the DDR, and its homeostasis was found to be regulated by histone PARylation factor 1 (HPF1), PARP1, and ARH3. HPF1 acts as an accessory factor in complex with PARP1/2 to specifically mono-ADP-ribosylate serine residues on histones and target proteins, while ARH3 functions to reverse this modification.[28,51,52] The finding that 14 out of the 16 ADP-ribosylation sites mutated in NuMA^{PARmut} are serine residues suggests that their ADP-ribosylation could be influenced by HPF1, PARP1, and ARH3. This hypothesis is supported by a proteome-wide scale mass spectrometry study that identified 6 out of the 14 serine residues (highlighted in yellow in Table S1) in NuMA as ADP-ribosylated, with this modification being dependent on HPF1.[53]

It is known that PARP1 detects SSBs and then recruits XRCC1, which acts as a scaffold protein to facilitate the recruitment of PNKP, APTX, and DNA ligase 3 to repair the SSB.[54–57] Moreover, PARP1 recruits and activates TDP1 to cleave TOP1ccs that are formed due to abortive TOP1 activity before being repaired subsequently by the SSBR machinery.[41,55] The fact that ADP-ribosylation of NuMA is required to facilitate its interaction with TDP1, but not with PARP1 or XRCC1, suggests that NuMA could be functioning downstream of the break formation. In other words, upon the formation of an SSB, PARP1 senses the SSB and is recruited to the break site. PARP1 then ADP-ribosylates itself as well as other repair proteins, including NuMA and TDP1. Next, NuMA functions to facilitate the recruitment of TDP1 to the SSB site to perform their repair function (Figure 6). This is also in line with the findings that in the absence of NuMA, there is a reduction in the enrichment of TDP1 at promoters of *FOS*, *SRF*, and *CCN2*,[18] which, as reported here, are sites of oxidative DNA breaks. This suggests that NuMA facilitates the recruitment of TDP1 to the damage sites. These results underscore the intricate interplay between ADP-ribosylation and protein function in coordinating cellular responses to DNA damage.

It is worth noting that while the global chromatin enrichment of TDP1 in both WT and NuMA-deficient cells is unaltered, it does not specifically reflect its recruitment to SSB sites. In fact, given that the *FOS*, *SRF*, and *CCN2* promoters are enriched with SSBs, TDP1 recruitment to these sites was previously reported to be NuMA dependent,[18] and we now report that this is also ADP-ribosylation dependent. This suggests that although the global chromatin association of TDP1 remains unchanged, its site-specific recruitment to SSBs is facilitated by NuMA.

Our findings also reveal the impact of NuMA ADP-ribosylation on regulating the transcription of NRGs (including IERGs) following oxidative stress. NuMA has been implicated in promoting the expression of the NRGs, including IERGs.[18] We demonstrate that NuMA ADP-ribosylation is important for modulating gene expression following oxidative stress. The decreased expression of the NRGs in cells complemented with NuMA^{PARmut} suggests a transcriptional defect when the ADP-ribosylation of NuMA is perturbed, underscoring the significance of this PTM in modulating gene expression patterns in response to oxidative stress. This aligns with our finding that NuMA could be acting as a PAR sink that is ADP-ribosylated in response to oxidative stress to limit the ADP-ribosylation of RNAPII,

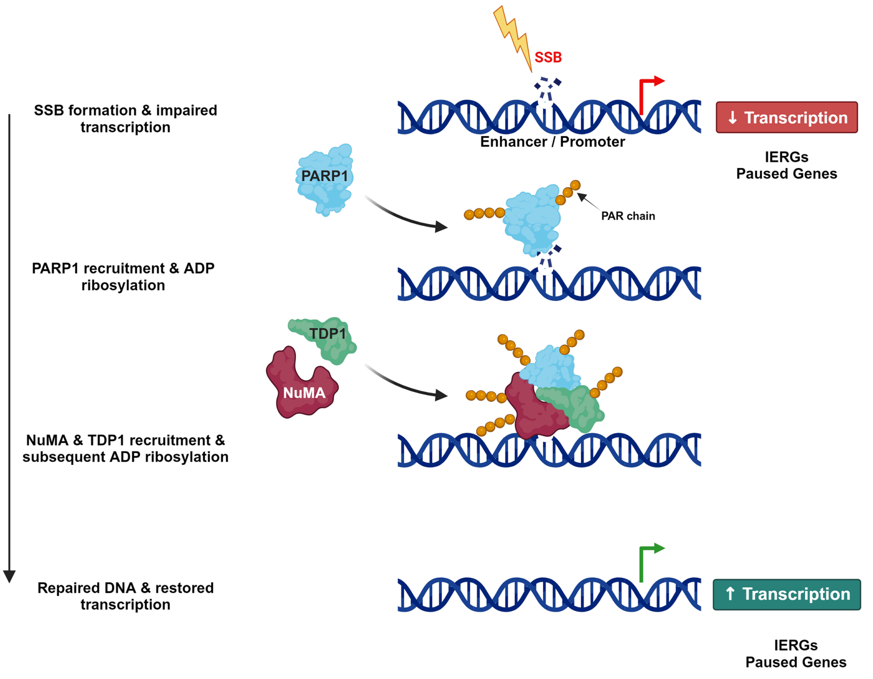

Figure 6. Model depicting the role of ADP-ribosylation in promoting the roles of NuMA in repair and transcription

Upon the formation of an SSB, transcription of immediate-early response genes (IERGs) and paused genes is impaired. PARP1 is then recruited to the break site, where it is ADP-ribosylated. It then ADP-ribosylates other repair proteins such as NuMA and TDP1, facilitating their recruitment to the SSB site to repair the SSB. Once repaired, transcription is restored.

increasing its availability at the chromatin to promote transcription.[18] It is also in line with reports suggesting that ADP-ribosylation of transcription factors and co-regulators such as NELF-E,[58] Sp1,[59] Oct-1,[60] and hnRNPs[61] affects the transcription profiles in cells.

The H_2O_2 treatment protocol used in this study provides physiologically relevant conditions of transient oxidative stress, which can arise during normal cellular metabolism and have an impact on transcriptional regulation. Our results show that the role of NuMA in regulating SSBR and transcription extends beyond exogenous oxidative stress because under unperturbed conditions, NuMA-deficient and $NuMA^{PARmut}$-expressing cells showed higher levels of DNA damage by alkaline comet assay and accumulated higher levels of oxidative DNA breaks at promoters and enhancers. Consistently, they also exhibited reduced transcription levels of NRGs, suggesting that NuMA ADP-ribosylation affects transcription regulation in response to both endogenous and exogenous sources of oxidative stress.

Overall, this study provides insights into the role of NuMA ADP-ribosylation in mediating DNA repair and transcriptional regulation in response to oxidative stress. Future studies elucidating the specific molecular mechanisms underlying NuMA ADP-ribosylation, the kinetics of ADP-ribosylation during the repair process, and the functional consequences will further enhance our understanding of the impact of this specific PTM in governing cellular responses to oxidative stress.

Additionally, investigating the potential crosstalk between NuMA and other PTMs such as phosphorylation and ubiquitination in modulating the DDR will be of great interest, particularly since they are highly integrated and dependent on one another. Out of the 16 ADP-ribosylation sites reported for NuMA, 8 have also been reported to be susceptible to phosphorylation, which impacts the role of NuMA in spindle maintenance.[62,63] Of note, none of those serine residues has a reported role in SSBR or transcription. Further research would be required to identify whether the phosphorylation of NuMA at the ADP-ribosylation sites as well as the other reported sites, particularly those at the C terminus globular domain,[64–68] are required for its role in SSBR and transcription regulation in response to oxidative stress.

Limitations of the study

While this study focuses on the impact of ADP-ribosylation-deficient mutations in specific residues in NuMA, there could be other residues that were not identified in the mass spectrometry study that were not mutated, which may contribute to the DNA repair and transcriptional roles investigated in this study. The conclusions are therefore limited to the specific sites that have been mutated. Additionally, assessing the effect of the ADP-ribosylation defect of NuMA in SSBR was largely locus -specific.

A further limitation is the potential structural consequences of the amino acid substitutions introduced in $NuMA^{PARmut}$. While structural modeling using AlphaFold suggests that there were no detectable differences in the structure of $NuMA^{WT}$ and $NuMA^{PARmut}$, we cannot entirely exclude the possibility that subtle changes in DNA-binding affinity or protein-protein interactions may influence the role of NuMA in mediating oxidative break repair and transcription. Although our findings support the conclusion that the observed phenotypic differences primarily result from the loss of ADP-ribosylation, we acknowledge the inherent limitations of computational modeling and the possibility that structural variations could contribute to the functional differences observed between $NuMA^{WT}$ and $NuMA^{PARmut}$. Further structural and biophysical analyses would be required to fully resolve these potential effects.

RESOURCE AVAILABILITY

Lead contact

Further information and requests for resources and reagents should be directed to and will be fulfilled by the lead contact, Sherif El-Khamisy (s.el-khamisy@bradford.ac.uk).

Materials availability

All unique plasmids generated in the study are available from the lead contact with a completed materials transfer agreement.

Data and code availability

- All data needed to evaluate the conclusions in the paper are present in the supplemental information.
- Original uncropped western blot images have been included in the supplemental figures.
- This study did not generate any custom code or genomics data.
- Any additional information required to reanalyze the data reported in this paper is available from the lead contact upon request.

ACKNOWLEDGMENTS

This work is supported by awards from the Lister Institute of Preventive Medicine (137661), the Medical Research Council (MR/Y000021/1), and the Wellcome Trust (103844) to S.F.E.-K. S.A. is funded by a scholarship from the Ministry of Higher Education of the Arab Republic of Egypt (MM13/21). M.D. is the recipient of a studentship jointly funded by the A Star Institute Singapore and the University of Sheffield. The graphical abstract was created using BioRender (https://BioRender.com/q35l917).

AUTHOR CONTRIBUTIONS

A.A.A. performed the IP, comet, OGG1-AP-qPCR, qPCR, and ChIP-qPCR experiments. C.L. performed the cloning, IP, and comet experiments. S.A. assisted with the OGG1-AP-qPCR and qPCR experiments. M.D. conducted the structure prediction using AlphaFold. A.A.A. and S.F.E.-K. wrote the manuscript. All authors edited the manuscript. S.F.E.-K. conceived the study and led and managed the project.

DECLARATION OF INTERESTS

The authors declare no competing interests.

STAR★METHODS

Detailed methods are provided in the online version of this paper and include the following:

- KEY RESOURCES TABLE
- EXPERIMENTAL MODEL AND STUDY PARTICIPANT DETAILS
  - Cell culture
- METHOD DETAILS
  - Hydrogen peroxide treatment
  - Recovery in serum-containing media
  - Generation of ADP-ribosylation-deficient NuMA construct (NuMA-^{PARmut})
  - Generation of corrected NuMA construct (NuMA^{Corrected})
  - siRNA-mediated depletion of NuMA in MRC5 cells
  - Plasmid transfection
  - Generation of whole cell extract
  - Cell fractionation
  - GFP Co-immunoprecipitation
  - Western blotting
  - Alkaline comet assay
  - OGG1-AP-qPCR
  - Quantitative polymerase chain reaction (qPCR)
  - ChIP-qPCR
  - AlphaFold structural predictions
- QUANTIFICATION AND STATISTICAL ANALYSIS

SUPPLEMENTAL INFORMATION

Supplemental information can be found online at https://doi.org/10.1016/j.celrep.2025.115737.

Received: July 24, 2024
Revised: March 3, 2025
Accepted: May 2, 2025
Published: May 20, 2025

REFERENCES

1. Lindahl, T., and Barnes, D.E. (2000). Repair of Endogenous DNA Damage. Cold Spring Harb. Symp. Quant. Biol. *65*, 127–133. https://doi.org/10.1101/sqb.2000.65.127.
2. Jackson, S.P., and Bartek, J. (2009). The DNA-damage response in human biology and disease. Nature *461*, 1071–1078. https://doi.org/10.1038/nature08467.
3. Caldecott, K.W. (2022). DNA single-strand break repair and human genetic disease. Trends Cell Biol. *32*, 733–745. https://doi.org/10.1016/j.tcb.2022.04.010.
4. Lindahl, T. (1993). Instability and decay of the primary structure of DNA. Nature *362*, 709–715. https://doi.org/10.1038/362709a0.
5. Demple, B., and Harrison, L. (1994). REPAIR OF OXIDATIVE DAMAGE TO DNA: Enzymology and Biology. Annu. Rev. Biochem. *63*, 915–948. https://doi.org/10.1146/annurev.bi.63.070194.004411.
6. Wei, X., Wang, Z., Hinson, C., and Yang, K. (2022). Human TDP1, APE1 and TREX1 repair 3′-DNA–peptide protein cross-links arising from abasic sites in vitro. Nucleic Acids Res. *50*, 3638–3657. https://doi.org/10.1093/nar/gkac185.
7. Sengupta, S., Wang, H., Yang, C., Szczesny, B., Hegde, M.L., and Mitra, S. (2020). Ligand-induced gene activation is associated with oxidative genome damage whose repair is required for transcription. Proc. Natl. Acad. Sci. USA *117*, 22183–22192. https://doi.org/10.1073/pnas.1919445117.
8. Afanas'ev, I. (2015). Mechanisms of Superoxide Signaling in Epigenetic Processes: Relation to Aging and Cancer. Aging Dis. *6*, 216–227. https://doi.org/10.14336/AD.2014.0924.
9. David, S.S., O'Shea, V.L., and Kundu, S. (2007). Base-excision repair of oxidative DNA damage. Nature *447*, 941–950. https://doi.org/10.1038/nature05978.
10. Burrows, C.J., and Muller, J.G. (1998). Oxidative Nucleobase Modifications Leading to Strand Scission. Chem. Rev. *98*, 1109–1152. https://doi.org/10.1021/cr960421s.
11. Niu, Y., DesMarais, T.L., Tong, Z., Yao, Y., and Costa, M. (2015). Oxidative stress alters global histone modification and DNA methylation. Free Radic. Biol. Med. *82*, 22–28. https://doi.org/10.1016/j.freeradbiomed.2015.01.028.
12. Hosseini, A., and Minucci, S. (2017). A comprehensive review of lysine-specific demethylase 1 and its roles in cancer. Epigenomics *9*, 1123–1142. https://doi.org/10.2217/epi-2017-0022.
13. Kooistra, S.M., and Helin, K. (2012). Molecular mechanisms and potential functions of histone demethylases. Nat. Rev. Mol. Cell Biol. *13*, 297–311. https://doi.org/10.1038/nrm3327.
14. Caldecott, K.W. (2007). Mammalian single-strand break repair: Mechanisms and links with chromatin. DNA Repair *6*, 443–453. https://doi.org/10.1016/j.dnarep.2006.10.006.
15. Caldecott, K.W. (2003). XRCC1 and DNA strand break repair. DNA Repair *2*, 955–969. https://doi.org/10.1016/S1568-7864(03)00118-6.

16. Lan, L., Nakajima, S., Oohata, Y., Takao, M., Okano, S., Masutani, M., Wilson, S.H., and Yasui, A. (2004). In situ analysis of repair processes for oxidative DNA damage in mammalian cells. Proc. Natl. Acad. Sci. USA *101*, 13738–13743. https://doi.org/10.1073/pnas.0406048101.

17. Mortusewicz, O., Rothbauer, U., Cardoso, M.C., and Leonhardt, H. (2006). Differential recruitment of DNA Ligase I and III to DNA repair sites. Nucleic Acids Res. *34*, 3523–3532. https://doi.org/10.1093/nar/gkl492.

18. Ray, S., Abugable, A.A., Parker, J., Liversidge, K., Palminha, N.M., Liao, C., Acosta-Martin, A.E., Souza, C.D.S., Jurga, M., Sudbery, I., and El-Khamisy, S.F. (2022). A mechanism for oxidative damage repair at gene regulatory elements. Nature *609*, 1038–1047. https://doi.org/10.1038/s41586-022-05217-8.

19. Chang, W., Dynek, J.N., and Smith, S. (2005). NuMA is a major acceptor of poly(ADP-ribosyl)ation by tankyrase 1 in mitosis. Biochem. J. *391*, 177–184. https://doi.org/10.1042/BJ20050885.

20. Salvador Moreno, N., Liu, J., Haas, K.M., Parker, L.L., Chakraborty, C., Kron, S.J., Hodges, K., Miller, L.D., Langefeld, C., Robinson, P.J., et al. (2019). The nuclear structural protein NuMA is a negative regulator of 53BP1 in DNA double-strand break repair. Nucleic Acids Res. *47*, 2703–2715. https://doi.org/10.1093/nar/gkz138.

21. Boehler, C., Gauthier, L.R., Mortusewicz, O., Biard, D.S., Saliou, J.-M., Bresson, A., Sanglier-Cianferani, S., Smith, S., Schreiber, V., Boussin, F., and Dantzer, F. (2011). Poly(ADP-ribose) polymerase 3 (PARP3), a newcomer in cellular response to DNA damage and mitotic progression. Proc. Natl. Acad. Sci. USA *108*, 2783–2788. https://doi.org/10.1073/pnas.1016574108.

22. Martello, R., Leutert, M., Jungmichel, S., Bilan, V., Larsen, S.C., Young, C., Hottiger, M.O., and Nielsen, M.L. (2016). Proteome-wide identification of the endogenous ADP-ribosylome of mammalian cells and tissue. Nat. Commun. *7*, 12917. https://doi.org/10.1038/ncomms12917.

23. Altmeyer, M., Messner, S., Hassa, P.O., Fey, M., and Hottiger, M.O. (2009). Molecular mechanism of poly(ADP-ribosyl)ation by PARP1 and identification of lysine residues as ADP-ribose acceptor sites. Nucleic Acids Res. *37*, 3723–3738. https://doi.org/10.1093/nar/gkp229.

24. Laing, S., Unger, M., Koch-Nolte, F., and Haag, F. (2011). ADP-ribosylation of arginine. Amino Acids *41*, 257–269. https://doi.org/10.1007/s00726-010-0676-2.

25. Zhang, Y., Wang, J., Ding, M., and Yu, Y. (2013). Site-specific characterization of the Asp- and Glu-ADP-ribosylated proteome. Nat. Methods *10*, 981–984. https://doi.org/10.1038/nmeth.2603.

26. Palazzo, L., Suskiewicz, M.J., and Ahel, I. (2021). Serine ADP-ribosylation in DNA-damage response regulation. Curr. Opin. Genet. Dev. *71*, 106–113. https://doi.org/10.1016/j.gde.2021.07.005.

27. Palazzo, L., Leidecker, O., Prokhorova, E., Dauben, H., Matic, I., and Ahel, I. (2018). Serine is the major residue for ADP-ribosylation upon DNA damage. Elife *7*, e34334. https://doi.org/10.7554/eLife.34334.

28. Bonfiglio, J.J., Fontana, P., Zhang, Q., Colby, T., Gibbs-Seymour, I., Atanassov, I., Bartlett, E., Zaja, R., Ahel, I., and Matic, I. (2017). Serine ADP-Ribosylation Depends on HPF1. Mol. Cell *65*, 932–940.e6. https://doi.org/10.1016/j.molcel.2017.01.003.

29. Hendriks, I.A., Larsen, S.C., and Nielsen, M.L. (2019). An Advanced Strategy for Comprehensive Profiling of ADP-ribosylation Sites Using Mass Spectrometry-based Proteomics. Mol. Cell. Proteomics *18*, 1010–1026. https://doi.org/10.1074/mcp.TIR119.001315.

30. Jumper, J., Evans, R., Pritzel, A., Green, T., Figurnov, M., Ronneberger, O., Tunyasuvunakool, K., Bates, R., Žídek, A., Potapenko, A., et al. (2021). Highly accurate protein structure prediction with AlphaFold. Nature *596*, 583–589. https://doi.org/10.1038/s41586-021-03819-2.

31. Abramson, J., Adler, J., Dunger, J., Evans, R., Green, T., Pritzel, A., Ronneberger, O., Willmore, L., Ballard, A.J., Bambrick, J., et al. (2024). Accurate structure prediction of biomolecular interactions with AlphaFold 3. Nature *630*, 493–500. https://doi.org/10.1038/s41586-024-07487-w.

32. Brown, C.J., Takayama, S., Campen, A.M., Vise, P., Marshall, T.W., Oldfield, C.J., Williams, C.J., and Dunker, A.K. (2002). Evolutionary rate heterogeneity in proteins with long disordered regions. J. Mol. Evol. *55*, 104–110. https://doi.org/10.1007/s00239-001-2309-6.

33. Harborth, J., Wang, J., Gueth-Hallonet, C., Weber, K., and Osborn, M. (1999). Self assembly of NuMA: multiarm oligomers as structural units of a nuclear lattice. EMBO J. *18*, 1689–1700. https://doi.org/10.1093/emboj/18.6.1689.

34. Abugable, A.A., Antar, S., and El-Khamisy, S.F. (2024). Chromosomal single-strand break repair and neurological disease: Implications on transcription and emerging genomic tools. DNA Repair *135*, 103629. https://doi.org/10.1016/j.dnarep.2024.103629.

35. Reid, D.A., Reed, P.J., Schlachetzki, J.C.M., Nitulescu, I.I., Chou, G., Tsui, E.C., Jones, J.R., Chandran, S., Lu, A.T., McClain, C.A., et al. (2021). Incorporation of a nucleoside analog maps genome repair sites in postmitotic human neurons. Science *372*, 91–94. https://doi.org/10.1126/science.abb9032.

36. Liang, L., Cao, C., Ji, L., Cai, Z., Wang, D., Ye, R., Chen, J., Yu, X., Zhou, J., Bai, Z., et al. (2023). Complementary Alu sequences mediate enhancer–promoter selectivity. Nature *619*, 868–875. https://doi.org/10.1038/s41586-023-06323-x.

37. Wu, W., Kargbo-Hill, S.E., Nathan, W.J., Paiano, J., Callen, E., Wang, D., Shinoda, K., van Wietmarschen, N., Colón-Mercado, J.M., Zong, D., et al. (2021). Neuronal enhancers are hotspots for DNA single-strand break repair. Nature *593*, 440–444. https://doi.org/10.1038/s41586-021-03468-5.

38. Prokhorova, E., Zobel, F., Smith, R., Zentout, S., Gibbs-Seymour, I., Schützenhofer, K., Peters, A., Groslambert, J., Zorzini, V., Agnew, T., et al. (2021). Serine-linked PARP1 auto-modification controls PARP inhibitor response. Nat. Commun. *12*, 4055. https://doi.org/10.1038/s41467-021-24361-9.

39. Yang, C.-S., Jividen, K., Kamata, T., Dworak, N., Abbas, T., Oostdyk, L., Remlein, B., Pourfarjam, Y., Kim, I.K., Du, K.P., et al. (2021). Androgen signaling uses a writer and a reader of ADP-ribosylation to regulate protein complex assembly. Nat. Commun. *12*, 2705. https://doi.org/10.1038/s41467-021-23055-6.

40. Rakhimova, A., Ura, S., Hsu, D.-W., Wang, H.-Y., Pears, C.J., and Lakin, N. D. (2017). Site-specific ADP-ribosylation of histone H2B in response to DNA double strand breaks. Sci. Rep. *7*, 43750. https://doi.org/10.1038/srep43750.

41. Das, B.B., Huang, S.y.N., Murai, J., Rehman, I., Amé, J.-C., Sengupta, S., Das, S.K., Majumdar, P., Zhang, H., Biard, D., et al. (2014). PARP1-TDP1 coupling for the repair of topoisomerase I-induced DNA damage. Nucleic Acids Res. *42*, 4435–4449. https://doi.org/10.1093/nar/gku088.

42. Das, B.B., Antony, S., Gupta, S., Dexheimer, T.S., Redon, C.E., Garfield, S., Shiloh, Y., and Pommier, Y. (2009). Optimal function of the DNA repair enzyme TDP1 requires its phosphorylation by ATM and/or DNA-PK. EMBO J. *28*, 3667–3680. https://doi.org/10.1038/emboj.2009.302.

43. Chiang, S.-C., Carroll, J., and El-Khamisy, S.F. (2010). TDP1 serine 81 promotes interaction with DNA ligase IIIα and facilitates cell survival following DNA damage. Cell Cycle *9*, 588–595. https://doi.org/10.4161/cc.9.3.10598.

44. Hudson, J.J.R., Chiang, S.-C., Wells, O.S., Rookyard, C., and El-Khamisy, S.F. (2012). SUMO modification of the neuroprotective protein TDP1 facilitates chromosomal single-strand break repair. Nat. Commun. *3*, 733. https://doi.org/10.1038/ncomms1739.

45. Rehman, I., Basu, S.M., Das, S.K., Bhattacharjee, S., Ghosh, A., Pommier, Y., and Das, B.B. (2018). PRMT5-mediated arginine methylation of TDP1 for the repair of topoisomerase I covalent complexes. Nucleic Acids Res. *46*, 5601–5617. https://doi.org/10.1093/nar/gky291.

46. Liao, C., Beveridge, R., Hudson, J.J.R., Parker, J.D., Chiang, S.-C., Ray, S., Ashour, M.E., Sudbery, I., Dickman, M.J., and El-Khamisy, S.F. (2018). UCHL3 Regulates Topoisomerase-Induced Chromosomal Break

Repair by Controlling TDP1 Proteostasis. Cell Rep. *23*, 3352–3365. https://doi.org/10.1016/j.celrep.2018.05.033.

47. Bhattacharjee, S., Rehman, I., Nandy, S., and Das, B.B. (2022). Post-translational regulation of Tyrosyl-DNA phosphodiesterase (TDP1 and TDP2) for the repair of the trapped topoisomerase-DNA covalent complex. DNA Repair *111*, 103277. https://doi.org/10.1016/j.dnarep.2022.103277.

48. Chowdhuri, S.P., and Das, B.B. (2021). Top1-PARP1 association and beyond: from DNA topology to break repair. NAR Cancer *3*, zcab003. https://doi.org/10.1093/narcan/zcab003.

49. Coskuner-Weber, O., and Uversky, V.N. (2019). Alanine Scanning Effects on the Biochemical and Biophysical Properties of Intrinsically Disordered Proteins: A Case Study of the Histidine to Alanine Mutations in Amyloid-β42. J. Chem. Inf. Model. *59*, 871–884. https://doi.org/10.1021/acs.jcim.8b00926.

50. Jensen, M.R., Communie, G., Ribeiro, E.A., Martinez, N., Desfosses, A., Salmon, L., Mollica, L., Gabel, F., Jamin, M., Longhi, S., et al. (2011). Intrinsic disorder in measles virus nucleocapsids. Proc. Natl. Acad. Sci. USA *108*, 9839–9844. https://doi.org/10.1073/pnas.1103270108.

51. Gibbs-Seymour, I., Fontana, P., Rack, J.G.M., and Ahel, I. (2016). HPF1/C4orf27 Is a PARP-1-Interacting Protein that Regulates PARP-1 ADP-Ribosylation Activity. Mol. Cell *62*, 432–442. https://doi.org/10.1016/j.molcel.2016.03.008.

52. Fontana, P., Bonfiglio, J.J., Palazzo, L., Bartlett, E., Matic, I., and Ahel, I. (2017). Serine ADP-ribosylation reversal by the hydrolase ARH3. Elife *6*, e28533. https://doi.org/10.7554/eLife.28533.

53. Hendriks, I.A., Buch-Larsen, S.C., Prokhorova, E., Elsborg, J.D., Rebak, A. K.L.F.S., Zhu, K., Ahel, D., Lukas, C., Ahel, I., and Nielsen, M.L. (2021). The regulatory landscape of the human HPF1- and ARH3-dependent ADP-ribosylome. Nat. Commun. *12*, 5893. https://doi.org/10.1038/s41467-021-26172-4.

54. El-Khamisy, S.F., Masutani, M., Suzuki, H., and Caldecott, K.W. (2003). A requirement for PARP-1 for the assembly or stability of XRCC1 nuclear foci at sites of oxidative DNA damage. Nucleic Acids Res. *31*, 5526–5533. https://doi.org/10.1093/nar/gkg761.

55. Ray Chaudhuri, A., and Nussenzweig, A. (2017). The multifaceted roles of PARP1 in DNA repair and chromatin remodelling. Nat. Rev. Mol. Cell Biol. *18*, 610–621. https://doi.org/10.1038/nrm.2017.53.

56. Hanzlikova, H., Gittens, W., Krejcikova, K., Zeng, Z., and Caldecott, K.W. (2017). Overlapping roles for PARP1 and PARP2 in the recruitment of endogenous XRCC1 and PNKP into oxidized chromatin. Nucleic Acids Res. *45*, 2546–2557. https://doi.org/10.1093/nar/gkw1246.

57. Caldecott, K.W., McKeown, C.K., Tucker, J.D., Ljungquist, S., and Thompson, L.H. (1994). An interaction between the mammalian DNA repair protein XRCC1 and DNA ligase III. Mol. Cell Biol. *14*, 68–76. https://doi.org/10.1128/mcb.14.1.68-76.1994.

58. Awwad, S.W., Abu-Zhayia, E.R., Guttmann-Raviv, N., and Ayoub, N. (2017). NELF-E is recruited to DNA double-strand break sites to promote transcriptional repression and repair. EMBO Rep. *18*, 745–764. https://doi.org/10.15252/embr.201643191.

59. Zaniolo, K., Desnoyers, S., Leclerc, S., and Guérin, S.L. (2007). Regulation of poly(ADP-ribose) polymerase-1 (PARP-1) gene expression through the post-translational modification of Sp1: a nuclear target protein of PARP-1. BMC Mol. Biol. *8*, 96. https://doi.org/10.1186/1471-2199-8-96.

60. Nie, J. (1998). Interaction of Oct-1 and automodification domain of poly (ADP-ribose) synthetase. FEBS (Fed. Eur. Biochem. Soc.) Lett. *6*.

61. Gagne, J.-P., Hunter, J.M., Labrecque, B., Chabot, B., and Poirier, G.G. (2003). A proteomic approach to the identification of heterogeneous nuclear ribonucleoproteins as a new family of poly(ADP-ribose)-binding proteins. Biochem. J. *15*, 331–340. https://doi.org/10.1042/BJ20021675.

62. Kettenbach, A.N., Schweppe, D.K., Faherty, B.K., Pechenick, D., Pletnev, A.A., and Gerber, S.A. (2011). Quantitative Phosphoproteomics Identifies Substrates and Functional Modules of Aurora and Polo-Like Kinase Activities in Mitotic Cells. Sci. Signal. *4*, rs5. https://doi.org/10.1126/scisignal.2001497.

63. Gallini, S., Carminati, M., De Mattia, F., Pirovano, L., Martini, E., Oldani, A., Asteriti, I.A., Guarguaglini, G., and Mapelli, M. (2016). NuMA Phosphorylation by Aurora-A Orchestrates Spindle Orientation. Curr. Biol. *26*, 458–469. https://doi.org/10.1016/j.cub.2015.12.051.

64. Hadisurya, M., Li, L., Kuwaranancharoen, K., Wu, X., Lee, Z.-C., Alcalay, R.N., Padmanabhan, S., Tao, W.A., and Iliuk, A. (2023). Quantitative proteomics and phosphoproteomics of urinary extracellular vesicles define putative diagnostic biosignatures for Parkinson's disease19. Commun. Med. *3*, 64. https://doi.org/10.1038/s43856-023-00294-w.

65. Matsumura, S., Hamasaki, M., Yamamoto, T., Ebisuya, M., Sato, M., Nishida, E., and Toyoshima, F. (2012). ABL1 regulates spindle orientation in adherent cells and mammalian skin. Nat. Commun. *3*, 626. https://doi.org/10.1038/ncomms1634.

66. Sana, S., Keshri, R., Rajeevan, A., Kapoor, S., and Kotak, S. (2018). Plk1 regulates spindle orientation by phosphorylating NuMA in human cells. Life Sci. Alliance *1*, e201800223. https://doi.org/10.26508/lsa.201800223.

67. Xu, X., Duan, X., Lu, C., Lin, G., and Lu, G. (2011). Dynamic distribution of NuMA and microtubules in human fetal fibroblasts, developing oocytes and somatic cell nuclear transferred embryos. Hum. Reprod. *26*, 1052–1060. https://doi.org/10.1093/humrep/der067.

68. Kiyomitsu, T., and Cheeseman, I.M. (2013). Cortical Dynein and Asymmetric Membrane Elongation Coordinately Position the Spindle in Anaphase. Cell *154*, 391–402. https://doi.org/10.1016/j.cell.2013.06.010.

69. Poetsch, A.R., Boulton, S.J., and Luscombe, N.M. (2018). Genomic landscape of oxidative DNA damage and repair reveals regioselective protection from mutagenesis. Genome Biol. *19*, 215. https://doi.org/10.1186/s13059-018-1582-2.

70. Pettersen, E.F., Goddard, T.D., Huang, C.C., Meng, E.C., Couch, G.S., Croll, T.I., Morris, J.H., and Ferrin, T.E. (2021). UCSF ChimeraX: Structure visualization for researchers, educators, and developers. Protein Sci. *30*, 70–82. https://doi.org/10.1002/pro.3943.

CellPress
OPEN ACCESS

STAR★METHODS

KEY RESOURCES TABLE

REAGENT or RESOURCE	SOURCE	IDENTIFIER
Antibodies		
NuMA Antibody (F-11)	Santa Cruz Biotechnology	Cat. No.: sc-365532; RRID:AB_10846197
Myc-Tag (9B11) Mouse mAb	Cell Signaling Technology	Cat. No.: 2276S; RRID:AB_331783
PARP1 Antibody (F-2)	Santa Cruz Biotechnology	Cat. No.: sc-8007; RRID:AB_628105
Phospho-XRCC1 (Ser485, Thr488) Polyclonal Antibody	Bethyl	Cat. No.: A300-231A; RRID:AB_263347
Anti-poly(ADP-ribose), Clone 10H	Tulip Biolabs	Cat. No.: 1020/N; RRID:AB_2236736
Anti-GFP antibody	Abcam	Cat. No.: ab290; RRID:AB_303395
Mouse Anti-Actin, beta Monoclonal Antibody	Abcam	Cat. No.: ab8226; RRID:AB_306371
Anti RNA Polymerase II CTD monoclonal antibody (Clone MABI 0601)	2B Scientific	Cat. No.: MCA-MABI0601-100-EX-100UL; RRID: AB_2728735
Mouse IgG Isotype Control	Thermo Fisher	Cat. No.: 02–6502; RRID: AB_2532951
Goat Anti-Rabbit IgG (H + L)-HRP Conjugate	Bio-Rad Laboratories	Cat. No.: 170–6515; RRID:AB_11125142
Goat Anti-Mouse IgG (H + L)-HRP Conjugate	Bio-Rad Laboratories	Cat. No.: 170–6516; RRID: AB_11125547
Bacterial and virus strains		
NEB® 5-alpha Competent E. coli (High Efficiency)	New England Biolabs	Cat. No.: C2987
Chemicals, peptides, and recombinant proteins		
Hydrogen peroxide solution	Sigma Aldrich	Cat. No.: H1009
Metafectene Pro	Biontex	Cat. No.: T040
KOD Hot Start Polymerase	Sigma Aldrich	Cat. No.: 71086
PEI	Polysciences	Cat. No.: 23966-2
BaseMuncher Endonuclease	Expedeon	Cat. No.: BM0100
cOmplete EDTA-Free Protease Inhibitor Cocktail	Sigma Aldrich	Cat. No.: 11836170001
PhosSTOP	Sigma Aldrich	Cat. No.: 4906837001
ADP-HPD	Sigma Aldrich	Cat. No.: 118415
ChromoTek GFP-Trap Magnetic Particles M-270	Proteintech	Cat. No.: gtd-20
Clarity Western ECL Substrate	BioRad	Cat. No.: 1705061
Dynabeads MyOne Streptavidin T1	Invitrogen	Cat. No.: 65601
8-Oxoguanine DNA Glycosylase Human Recombinant	Prospec	Cat. No.: ENZ-253
Critical commercial assays		
QIAprep Spin Miniprep Kit	QIAgen	Cat. No.: 27104
Qiagen Blood and Tissue Kit	QIAgen	Cat. No.: 69504
MinElute Reaction Cleanup kit	QIAgen	Cat. No.: 28204
PreCR Repair Mix	QIAgen	Cat. No.: M0309L
QuantiNova SYBR Green PCR Kit	QIAgen	Cat. No.: 208057
RNeasy Plus Mini Kit	QIAgen	Cat. No.: 74134
QIAshredder	QIAgen	Cat. No.: 79654
cDNA Reverse Transcription Kit	Applied Biosystems	Cat. No.: 4368814
Experimental models: Cell lines		
MRC-5	ATCC	CCL-171
Oligonucleotides		
See Table S2 for list of primers and DNA sequences used in this study	Integrated DNA Technologies	N/A

(*Continued on next page*)

Continued

REAGENT or RESOURCE	SOURCE	IDENTIFIER
See Table S3 for list of siRNA sequences used in this study	Eurofins	N/A
Recombinant DNA		
pEGFP-C1 (EV)	Addgene	Cat. No.; 6082-1
pEGFP-C1-NuMA (GFP-NuMA^{WT})	Ray et al.[18]	N/A
GFP-NuMA^{PARmut}	This study	N/A
GFP-NuMA^{Corrected}	This study	N/A
pCI-neo-Myc-TDP1 (myc TDP1)	Hudson et al.[44]	N/A
Software and algorithms		
Comet Assay IV Software	Perceptive Instruments	RRID: N/A
GraphPad Prism 10	GraphPad Software	RRID:SCR_002798
Image Lab	Biorad	RRID:SCR_014210
Rotor-Gene Q Series Software	Qiagen	RRID:SCR_015740
AlphaFold	EMBL-EBI	RRID:SCR_023662

EXPERIMENTAL MODEL AND STUDY PARTICIPANT DETAILS

Cell culture

Normal human lung fibroblast MRC-5 cells were cultured in Minimum Essential Medium Eagle (MEM) supplemented with a final concentration of 10% fetal bovine serum, 1% penicillin/streptomycin and 1% L-glutamine at 37°C in a humidified atmosphere containing 5% carbon dioxide. Cells tested negative for mycoplasma.

METHOD DETAILS

Hydrogen peroxide treatment

Cells were washed with PBS then treated with freshly prepared 10 μM hydrogen peroxide (H_2O_2) in cold PBS for 10 min, on ice, in the dark. After treatment, the cells were washed again with cold PBS.

Recovery in serum-containing media

Following serum starvation and/or H_2O_2 treatment, cells were washed with PBS then incubated with media containing all the supplements including the serum for 90 min.

Generation of ADP-ribosylation-deficient NuMA construct (NuMA^{PARmut})

The mutations at positions 1609 and 2082 were incorporated in the GFP-NuMA^{WT} plasmid using overlapping PCR following the manufacturer's instructions for the KOD Hot Start DNA Polymerase (Cat. No.: 71086, Sigma Aldrich). The sequences of the primers used are described in Table S2. To introduce the mutation at position 1609 using the GFP-NuMA^{WT} plasmid as a template, the 1609_Fwd/AR and 1609_Rev/AF primer sets were used, followed by overlapping PCR with Primers AF/AR to generate Fragment A. To introduce the remaining 14 mutations, a DNA block of 867 bp (covering the region between 1794 and 2081) was synthesized. To introduce the mutation at position 2082, the DNA block was used as a template and the 2082_Fwd/BR and 2082_Rev/BF primer sets were used to introduce the mutation followed by overlapping PCR with Primers BF/BR to generate Fragment B. Overlapping PCR using Fragments A and B as template with primers AF/BR generated Fragment C which contains all the mutations. Fragment C was then finally cloned into the GFP-NuMA^{WT} using the Pfl23II/EcoR1 restriction enzymes. The transformation was conducted in DH5α competent cells (NEB) following the manufacturer's protocol. DNA extraction and purification were done using the QIAprep Spin Miniprep Kit (Cat. No.: 27104, QIAgen). Sanger sequencing confirmed the correct incorporation of the mutations (Eurofins).

Generation of corrected NuMA construct (NuMA^{Corrected})

The correction of the mutation at position 1528 from Lysine to Glutamic Acid was conducted in the GFP-NuMA^{WT} plasmid using overlapping PCR following the manufacturer's instructions for the KOD Hot Start DNA Polymerase (Cat. No.: 71086, Sigma Aldrich). The sequences of the primers used are described in TableS2. To introduce the mutation at position 1528 using the GFP-NuMA^{WT} plasmid as a template, the AF2/1528_Rev and 1528_Fwd/BR primer sets were used. This was followed by overlapping PCR with primers AF2/AR to generate a DNA fragment that was cloned into the GFP-NuMA^{WT} using the Pfl23II/EcoR1 restriction enzymes. The transformation was conducted in DH5α competent cells (NEB) following the manufacturer's protocol. DNA extraction and purification

were done using the QIAprep Spin Miniprep Kit (Cat. No.: 27104, QIAgen). Sanger sequencing confirmed the correct incorporation of the mutations (Eurofins).

siRNA-mediated depletion of NuMA in MRC5 cells

Cells were seeded at an appropriate seeding density (150,000 for 6-well plate and 6,000,000 for a 15 cm plate). They were transfected with 20 μM siRNA on Day 2 and 4 using Metafectene Pro (Cat. No.: T040–1.0, Biontex) in a 1:1 ratio in Opti-MEM | Reduced Serum Media. The sequences of the siRNA oligonucleotides used is described in Table S3.

Plasmid transfection

Cells were seeded at an appropriate seeding density (150,000 for 6-well plate and 6,000,000 for a 15 cm plate). They were transfected with 0.5 μg or 5 μg of plasmid on Day 3 using the linear 25K PEI (Cat. No.: 23966-2, Polysciences) in a 1:2 plasmid:PEI ratio in Opti-MEM | Reduced Serum Media.

Generation of whole cell extract

For immunoblotting, cells were lysed directly in 1x protein loading buffer and heated at 95°C for 5 min. For immunoprecipitation, cells were lysed in 400 μL NP-40 Lysis Buffer (50 mM Tris-HCl pH 8.0, 150 mM NaCl, 1% NP-40) and incubated on ice for 45 min with occasional vortexing and then centrifuged at 20,000 x *g* for 15 min at 4°C. The supernatant was then transferred to a clean Eppendorf tube and used immediately or snap frozen in liquid nitrogen and stored at −80°C. The buffer was supplemented with 1:1000 BaseMuncher Endonuclease (Cat. No.: BM0100, Expedeon), cOmplete EDTA-Free Protease Inhibitor Cocktail (Cat. No.: 11836170001), PhosSTOP (Cat. No.: 4906837001) and 5 μM ADP-HPD (Cat. No.: 118415).

Cell fractionation

Cells were lysed for 10 min at room temperature on an orbital shaker in 1.5 mL hypotonic buffer (20 mM HEPES-KOH pH 8.0, 10 mM KCl, 20% glycerol, 1 mM $MgCl_2$ and 0.1% Triton X-100). The cells were then scraped in the buffer and incubated on ice for 10 min. The lysates were then centrifuged at 6400 rpm for 4 min at 4°C. The supernatant, containing the cytoplasmic fraction, was discarded and the nuclear pellet was washed once with 600 μL hypotonic buffer. The lysate was centrifuged at 6400 rpm for 4 min at 4°C and the supernatant was discarded. The nuclear pellet was then lysed in 100 μL hypertonic buffer (20 mM HEPES-KOH pH 8.0, 1 mM EDTA pH 8.0, 20% glycerol, 400 mM NaCl, 0.1% Triton X-100) for 20 min on ice with periodic vortexing, and then centrifuged at 13,500 rpm for 5 min at 4°C. The supernatant, which contains the soluble nuclear fraction was collected in a clean microcentrifuge tube and stored on ice. The pellet was washed once with 100 μL hypertonic buffer and centrifuged at 13500 rpm for 5 min at 4°C and the supernatant was discarded. The chromatin bound proteins were then isolated by resuspending the pellet in 100 μL insoluble buffer (20 mM Tris HCl pH 8.0, 150 mM NaCl, 1% SDS, 1% NP-40 and 10 mM Iodoacetamide) and incubated for 50 min at 4 °C at 1000 rpm. This was followed by the addition of 0.5 μL BaseMuncher Endonuclease (Cat. No.: BM0100, Expedeon) and incubated at 25°C for 15 min at 1000 rpm. The lysate was then centrifuged at 13500 rpm for 5 min at 4°C and the supernatant, containing the insoluble nuclear fraction, was transferred to a new microcentrifuge tube. All lysis buffers contained cOmplete EDTA-Free Protease Inhibitor Cocktail (Cat. No.: 11836170001), PhosSTOP (Cat. No.: 4906837001) and 5 μM ADP-HPD (Cat. No.: 118415). They lysates were either used immediately or snap-frozen in liquid nitrogen at stored at −80°C.

GFP Co-immunoprecipitation

The lysates were quantified and 5% of the lysate volume used was set aside as input. 25 μL of ChromoTek GFP-Trap Magnetic Particles M-270 (Cat. No.: gtd-20, Proteintech) were equilibrated by washing thrice with 1 mL ice-cold IP Dilution Buffer (16.7 mM Tris-HCl pH 7.4, 167 mM NaCl, 1.2 mM EDTA pH 8.0, 1.1% Triton X-100) and then resuspended in 25 μL of IP Dilution Buffer. To the beads, 900 μL of IP dilution buffer was added followed by 100 μL of the lysate and then incubated on a rotator for 2 h at 4°C. The beads were then washed once with IP Dilution Buffer and twice with GFP Wash Buffer (10 mM Tris-HCl pH 7.4, 150 mM NaCl, 0.5 mM EDTA pH 8.0, 0.05% NP-40) by resuspending the beads in the buffer then incubating it on a rotator at room temperature for 2 min before discarding the wash buffer. The co-immunoprecipitated samples were then eluted in 50 μL 1× Protein Loading Buffer at 95°C for 10 min with vortexing.

Western blotting

4-15% gradient gels were used for immunoblotting. Transfer was conducted using the Trans-Blot Turbo Transfer System (Cat. No.: 1704150, Bio-Rad) and nitrocellulose membranes. The membranes were blocked in 5% milk in TBS-T and the primary and secondary antibodies used were also diluted in 5% milk in TBS-T. The bands were visualized using the Clarity Western ECL Substrate (Cat. No.: 1705061, Bio-Rad) on the ChemiDoc MP Gel Photo Documentation System (Bio-Rad). The images of the uncropped blots are shown in Figure S8.

Alkaline comet assay

Approximately 30,000 MRC5 cells were either untreated or treated with 20 μM H_2O_2. H_2O_2-treated cells were left to recover in complete medium for 30 or 60 min at 37°C. A thin layer of 0.6% agarose was laid onto frosted slides. Cells were resuspended in ice-cold

PBS before being mixed with an equal volume of 1.2% low-gelling-temperature agarose, maintained at 42°C. Slides were then placed at 4°C to set. Cells were lysed in a pre-chilled lysis buffer (2.5 M NaCl, 10 mM Tris HCl, 100 mM EDTA pH 8.0, 1% Triton X-100, 1% DMSO; pH 10) for 1 h at 4°C, before submerging in pre-chilled alkaline electrophoresis buffer (50 mM NaOH, 1 mM EDTA, 1% DMSO) for 45 min at 4°C. Electrophoresis was performed at 12 V for 25 min in the dark at 4°C, followed by the addition of 400 mM Tris HCl pH 7 to neutralize. DNA was stained with SYBR Green (1:10000 in PBS) before measuring the average tail moments using Comet Assay IV software (Perceptive Instruments, UK).

OGG1-AP-qPCR

OGG1-AP-qPCR was conducted as previously reported.[69] Briefly, 7–10 μg genomic DNA from cells was extracted using the Qiagen Blood and Tissue Kit (Cat. No.: 69504, Qiagen) and eluted in 100 μL H_2O. DNA was digested with 1:1,000 dilution of 8-oxoguanine DNA glycosylase (OGG1) in 1× NEB-buffer 2, 1× BSA and incubated at 37°C for 1 h. DNA was precipitated using cold 100% ethanol and reconstituted in 90 μL PBS. DNA was labeled with 5 mM biotin-labelled aldehyde-reactive probe (ARP). Labeled DNA was transferred to 1.5-mL tube and precipitated with ice-cold ethanol (100%), washed with 70% ethanol, and reconstituted in 130 μL TE buffer pH 8. DNA was subsequently sheared to an average peak size of 300 bp on a Bioruptor Pico (Diagenode). 30 μL sheared DNA was kept aside as inputs. 100 μL MyOne Dynabeads (Invitrogen) was washed twice with 1 M NaCl in TE buffer, reconstituted in 100 μL 2 M NaCl in TE buffer and added to 100 μL of labeled DNA from above. Samples were rotated at room temperature for 10 h. DNA was eluted twice from the beads using 95% formamide and 10 mM EDTA for 10 min at 65°C in a total 100 μL volume. MinElute Reaction Cleanup kit (Cat. No.: 28204, Qiagen) was used for DNA purification, and DNA was eluted in 30 μL TE (3 × 10 μL elution). DNA was repaired using the PreCR Repair Mix (Cat. No.: M0309L, NEB) as per the manufacturers protocol. Repaired DNA was purified using MinElute Clean Up Kit and eluted in 13 μL mQ H_2O. The DNA was then diluted 1:10 and 700 nM primers were used with the QuantiNova SYBR Green PCR Kit (7500) (Cat. No.: 208057, QIAgen) following the manufacturer's instructions for the qPCR reactions. The sequences of the primers used are listed in Table S2.

Quantitative polymerase chain reaction (qPCR)

Cells were lysed using RLT buffer from the RNeasy Plus Mini Kit (Cat. No.: 74134, QIAgen), homogenized using QIAshredder (Cat. No.: 79654, QIAgen). 1 μg of the RNA was used for cDNA synthesis using the High Capacity cDNA Reverse Transcription Kit (Cat. No.: 4368814, Applied Biosystems). Pooled cDNA was used to construct a standard curve using 5-point 10-fold serial dilution. The cDNA samples were diluted 10-fold and 5 μL cDNA, 700 nM primers were used with the QuantiNova SYBR Green PCR Kit (7500) (Cat. No.: 208057, QIAgen) following the manufacturer's instructions. The sequences of the primers used are listed in Table S2.

ChIP-qPCR

Cells were seeded and transfected as described above, serum starved for 48 h then either untreated or treated with 10 μM H_2O_2 for 10 min and left to recover for 90 min in serum-containing H_2O_2-free media. Cells were then crosslinked with 1% paraformaldehyde for 10 min at room temperature. The crosslinking was quenched with 0.125 M glycine for 5 min at room temperature. Cells were washed twice with cold PBS and then scraped. Cell pellets were obtained and lysed in 5 pellet volumes of ChIP Lysis Buffer 1 (50 mM HEPES-KOH pH 7.5, 140 mM NaCl, 1 mM EDTA, 10% Glycerol, 0.5% NP-40, 0.25% Triton X-100) and incubated for 5 min in 4 °C at a rotator, followed by centrifugation at 3000 x *g* for 5 min at 4°C. The cell pellet was then resuspended in 5 pellet volumes of ChIP Buffer 2 (10 mM Tris HCl pH 7.4, 200 mM NaCl, 1 mM EDTA, 0.5 mM EGTA) and incubated on a rotator for 10 min at room temperature, followed by centrifugation at 1500 x *g* for 5 min at 4°C. The nuclear pellet was then lysed in a suitable volume of ChIP Lysis Buffer 3 (10 mM Tris HCl pH 7.4, 200 mM NaCl, 1 mM EDTA, 0.5 mM EGTA, 0.1% Sodium deoxycholate, 0.5% Sodium lauroylsarcosine) and sonicated using Bioruptor Pico to yield DNA fragments of the size 100–300 bp and cleared by centrifugation at 20000 x *g*, at 4°C for 15 min. For RNAPII ChIP-qPCR, lysates containing an equal quantity of DNA were incubated with either 4 μg anti-pan-RNAPII antibody or anti-Mouse IgG overnight at 4°C with 1% of the lysate reserved as an input. Next, 30 μL Protein G Dynabeads were added to each sample and incubated for 2 h at 4°C. For GFP-NuMA ChIP-qPCR, 25 μL of GFP-Trap beads were added to the lysate and incubated overnight at 4°C. The beads were washed once in Low Salt Wash Buffer (0.1% SDS, 1% Triton X-100, 2 mM EDTA, 20 mM Tris-HCl pH 8.0 and 150 mM NaCl), once in High Salt Wash Buffer (0.1% SDS, 1% Triton X-100, 2 mM EDTA, 20 mM Tris-HCl pH 8.0, 500 mM NaCl) and once in LiCl Wash Buffer (0.25M LiCl, 1% NP-40, 1% Sodium deoxycholate, 1 mM EDTA, 10 mM Tris-HCl pH 8.0) before being eluted twice in 100 μL ChIP Elution Buffer (50 mM Tris-HCl pH 8.0, 10 mM EDTA pH 8.0, 1% SDS) at 65°C for 30 min at 1000 rpm. The eluted DNA and the input were reverse crosslinked by incubating with up to 0.2 M NaCl at 65°C for 16 h. Treatment with 0.2 mg/mL RNase A was done at 37°C for 30 min at 800 rpm and 0.2 mg/mL Proteinase K at 55°C for 2 h at 8 rpm. DNA was purified by phenol chloroform extraction followed by ethanol precipitation. The eluted DNA (10 μL) was diluted 1:10 then subjected to qPCR using primers listed in Table S2 and the % input was calculated.

AlphaFold structural predictions

The structure of the C terminus globular domain of $NuMA^{WT}$ and $NuMA^{PARmut}$ was predicted using the modified version of AlphaFold v2.3.2 used in the AlphaFold.ipynb Colab notebook (https://colab.research.google.com/github/deepmind/alphafold/blob/main/notebooks/AlphaFold.ipynb#scrollTo=pc5-mbsX9PZC).[30] The remaining structures and structural interactions were predicted using

the Beta version of AlphaFold 3[31] hosted on the Google DeepMind server (https://golgi.sandbox.google.com/). The predicted structures were further visualized and analyzed using UCFS ChimeraX v1.4.[70]

QUANTIFICATION AND STATISTICAL ANALYSIS

GraphPad Prism 10 was used for conducting the statistical tests and generating the graphs described in this study. The statistical tests and number of repeats are described in the figure legends, the *p*-values are shown in the figure and the error bars represent the standard error of the mean or the range, as described in the figure legends. Statistical significance was defined as a two-sided *p*-value less than 0.05.

Cell Reports, Volume *44*

Supplemental information

ADP-ribosylation of NuMA promotes DNA single-strand break repair and transcription

Arwa A. Abugable, Chunyan Liao, Sarah Antar, Matthew Dowson, and Sherif F. El-Khamisy

ADP-ribosylation of NuMA promotes DNA single-strand break repair and transcription

Arwa A. Abugable[1,2,5], Chunyan Liao[1,2], Sarah Antar[1,2,3], Matthew Dowson[1,2], Sherif F. El-Khamisy[1,2,4,6,*]
[1]School of Biosciences, Firth Court, University of Sheffield, Sheffield, UK
[2]The Healthy Lifespan and Neuroscience Institutes, University of Sheffield, Sheffield, UK
[3]Medical Biochemistry and Molecular Biology Department, Faculty of Medicine, Mansoura University, Egypt
[4]Institute of Cancer Therapeutics, School of Pharmacy and Medical Sciences, University of Bradford, Bradford, UK
[5]Present address: Department of Biochemistry, University of Oxford, South Parks Road, Oxford, UK
[6]Lead Contact
*Correspondence: s.el-khamisy@bradford.ac.uk

Table of Contents

Figure S1: AlphaFold modelling of NuMA^{WT} and NuMA^{PARmut} (related to Figure 2) 2

Figure S2: Corrected mutation in NuMA^{WT} does not impair SSBR kinetics (related to Figure 4a, b) 3

Figure S3: Enrichment of NuMA at specific genomic loci (related to Figures 4c,d) 4

Figure S4: Transcript levels of NuMA-regulated genes (related to Figure 5) 4

Figure S5: Cold shock does not change transcript levels of NuMA-regulated genes (related to Figure 5) 5

Figure S6: Enrichment of RNAPII at specific genomic loci (related to Figure 5) 5

Figure S7: TDP1 over-expression does not rescue repair and transcription defect of NuMA^{PARmut} (related to Figures 4 and 5) 6

Figure S8: Uncropped blots (Related to Figures 1, 3 and S2) 7

Table S1: ADP-ribosylation sites that are mutated in NuMA^{PARmut} (related to Figure 2) 8

Table S2: List of primers used in this study 8

Table S3: List of siRNA sequences used in this study 10

References 10

Figure S1: AlphaFold modelling of NuMA^{WT} and NuMA^{PARmut} (related to Figure 2)

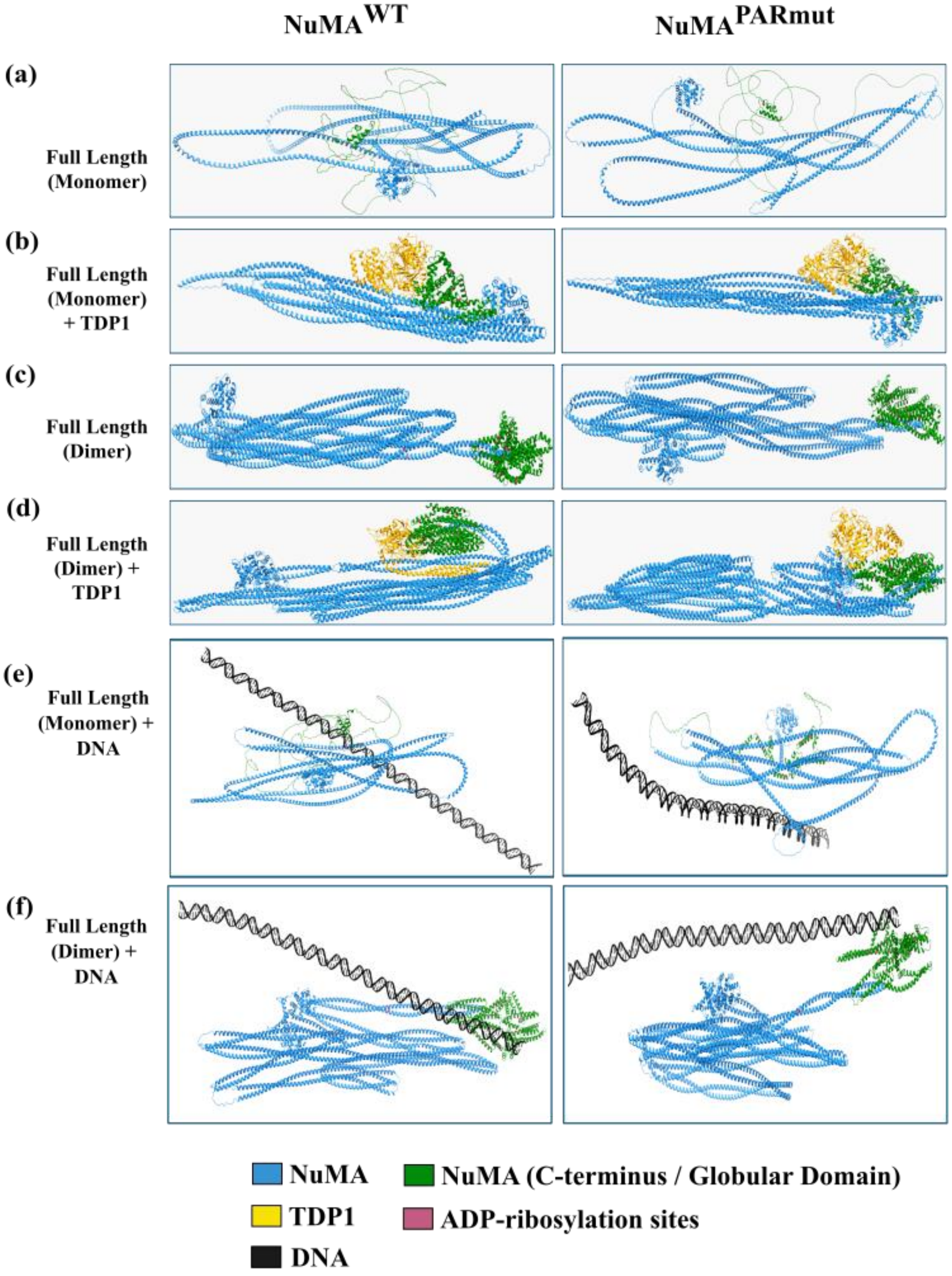

Figure S1: AlphaFold modelling of NuMA^{WT} and NuMA^{PARmut}

Predicted structures of (**a**) full length NuMA^{WT} and NuMA^{PARmut} as monomers and (**b**) in complex with TDP1, (**c**) full length NuMA^{WT} and NuMA^{PARmut} as dimers and (**d**) in complex with TDP1, (**e**) full length NuMA^{WT} and NuMA^{PARmut} as monomers in complex with DNA and (**f**) as dimers in complex with DNA.

Figure S2: Corrected mutation in NuMA^{WT} does not impair SSBR kinetics (related to Figure 4a, b)

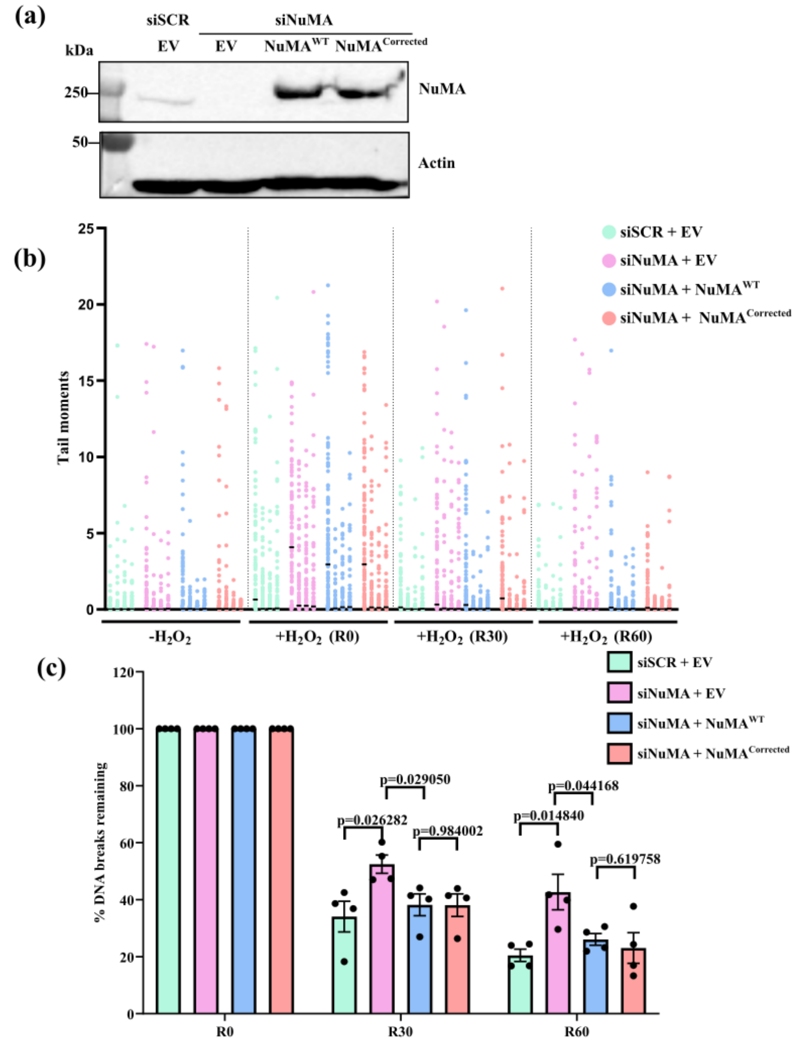

Figure S2: Corrected mutation in NuMA^{WT} does not impair SSBR kinetics

(**a**) Representative immunoblotting from whole cell extracts showing the successful knock down of endogenous NuMA with siRNA and transfection of the empty vector (EV), NuMA^{WT} and NuMA^{Corrected} constructs. (**b and c**) Cells were transfected with siSCR or siNuMA and then complemented with either EV, NuMA^{WT} or NuMA^{Corrected} constructs. The cells were left untreated or treated with 10 μM H_2O_2 for 10 minutes on ice in the dark, followed by recovery in H_2O_2-free media for 0, 30 and 60 minutes, denoted as R0, R30 and R60, respectively, before being subjected to alkaline comet assay. (**b**) Violin plot showing the distribution of the of comet tail moments at the indicated time points. The data shown are from 4 biological replicates. (**c**) Bar plot showing the % DNA breaks remaining during the recovery time points. The bar char represents data from 4 biological replicates with error bars representing the standard error of the mean. Two-sided unpaired Student t-test was conducted.

Figure S3: Enrichment of NuMA at specific genomic loci (related to Figures 4c,d)

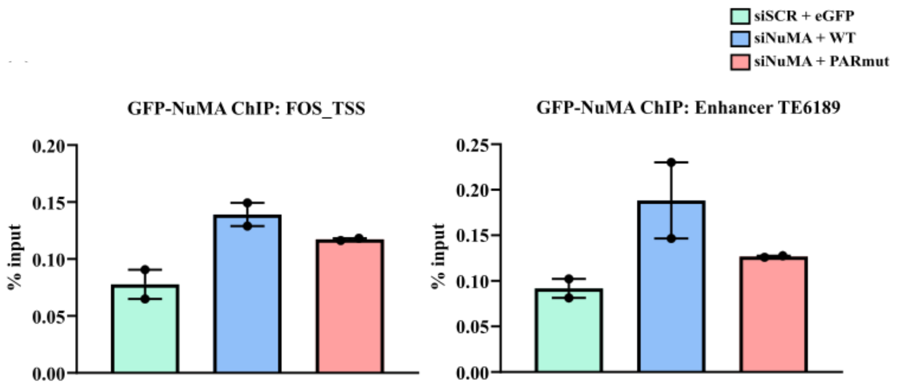

Figure S3: Enrichment of NuMA at specific genomic loci

Cells were transfected with siSCR or siNuMA and then complemented with either empty vector (EV), NuMA^{WT} or NuMA^{PARmut} constructs, serum-starved for 48 hours and then treated with 10 µM H_2O_2 for 10 minutes on ice in the dark, followed by a 90-minute recovery in serum-containing media. GFP-NuMA ChIP-qPCR was conducted at the *FOS* promoter and TE6189 enhancer. The bar chart shows the % input from 2 biological replicates with error bars representing the range.

Figure S4: Transcript levels of NuMA-regulated genes (related to Figure 5)

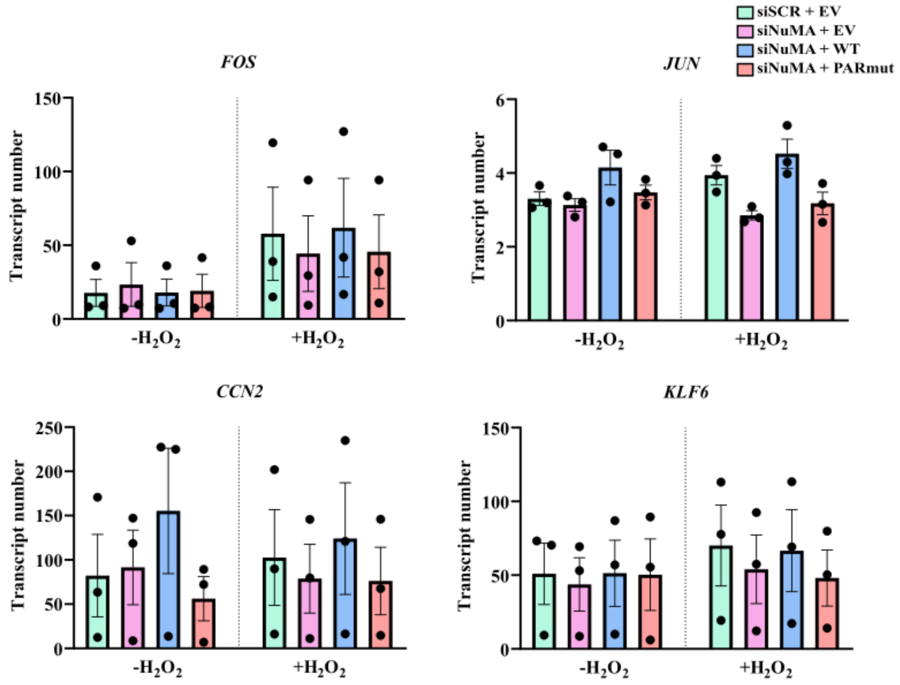

Figure S4: Transcript levels of NuMA-regulated genes

Cells were transfected with siSCR or siNuMA, complemented with EV, NuMA^{WT} or NuMA^{PARmut}, serum starved for 48 hours, treated with 10 µM H_2O_2 for 10 minutes on ice in the dark and recovered in serum-containing media for 90 minutes. Bar plots showing transcript number following qPCR of *FOS*, *JUN*, *CCN2* and *KLF6* (n=3 biological replicates, mean±SEM).

Figure S5: Cold shock does not change transcript levels of NuMA-regulated genes (related to Figure 5)

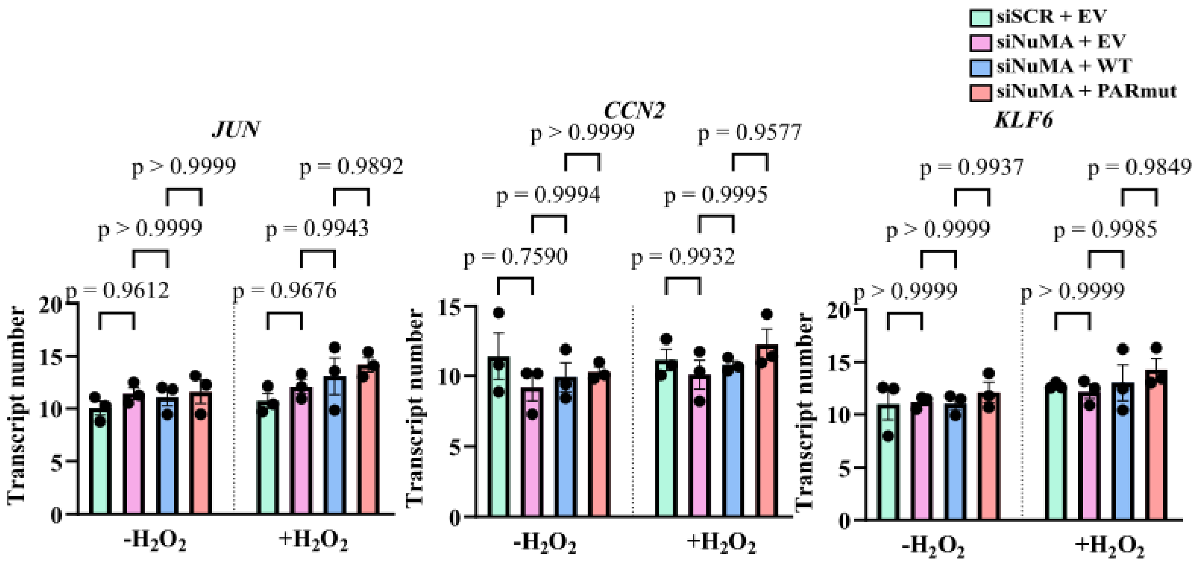

Figure S5: Cold shock does not change transcript levels of NuMA-regulated genes

Cells were transfected with siSCR or siNuMA and then complemented with either empty vector (EV), NuMA^{WT} or NuMA^{PARmut} constructs, serum-starved for 48 hours and then treated with 10 µM H_2O_2 in cold PBS (+H_2O_2) or just cold PBS (-H_2O_2) for 10 minutes on ice in the dark, followed by qPCR analysis for the expression of *JUN*, *CCN2* and *KLF6*. Bar plots showing transcript number (n=3 biological replicates, mean±SEM). One-way ANOVA was conducted.

Figure S6: Enrichment of RNAPII at specific genomic loci (related to Figure 5)

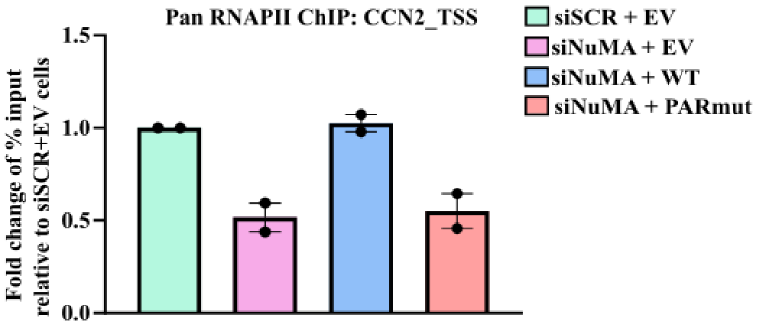

Figure S6: Enrichment of RNAPII at CCN2 promoter

Cells were transfected with siSCR or siNuMA and then complemented with either empty vector (EV), NuMA^{WT} or NuMA^{PARmut} constructs, serum-starved for 48 hours and then treated with 10 µM H_2O_2 for 10 minutes on ice in the dark, followed by a 90-minute recovery in serum-containing media. Pan RNAPII ChIP-qPCR was conducted at the *CCN2* promoter. The bar chart shows the fold change in % input relative to the siSCR+EV-transfected cells and represents data from 2 biological replicates with error bars representing the range.

Figure S7: TDP1 over-expression does not rescue repair and transcription defect of NuMA^{PARmut} (related to Figures 4 and 5)

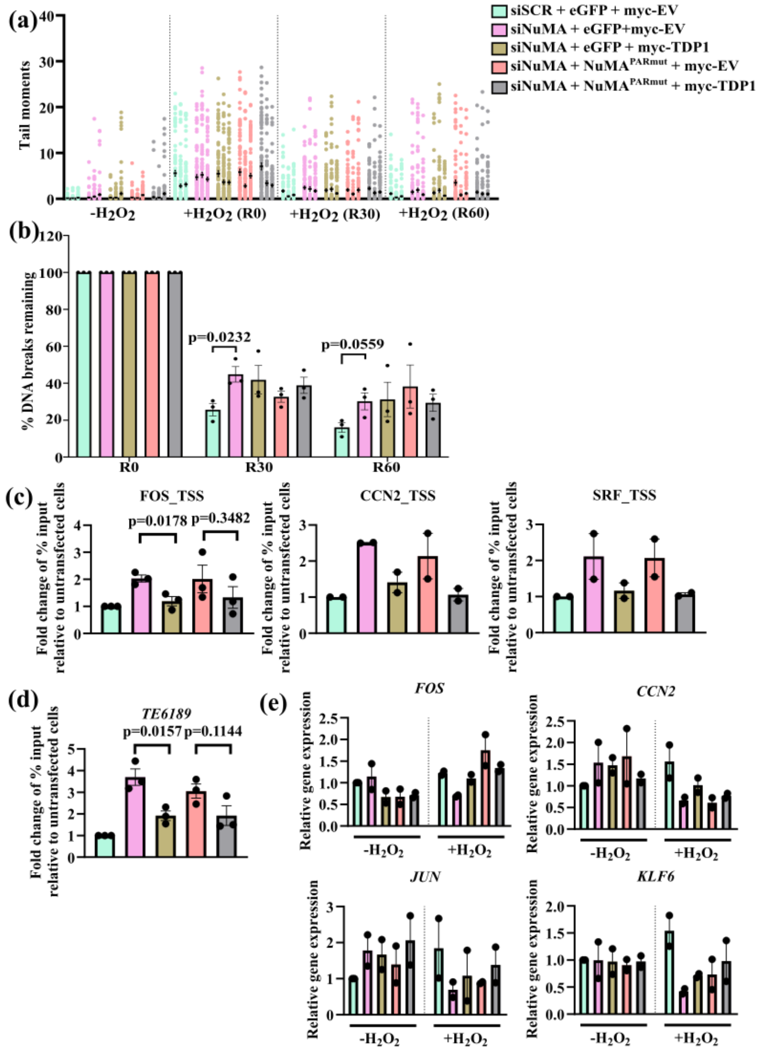

Figure S7: TDP1 over-expression does not rescue repair and transcription defect of NuMA^{PARmut}

(a-d) Cells transfected with siSCR or siNuMA and then complemented with either EV, NuMA^{WT} or NuMA^{PARmut}, and either myc-EV or myc-TDP1. (**a and b**) Cells were left untreated or treated with 20 µM H_2O_2 for 10 minutes on ice in the dark, followed by recovery in H_2O_2-free media for 0, 30 and 60 minutes (R0, R30 and R60) and used for alkaline comet assay. (**a**) Scatter plot of comet tail moments at the indicated time points. (**b**) Bar plot of % DNA breaks remaining (n=3 biological replicates, mean±SEM). Two-sided unpaired Student t-test was conducted. (**c-d**) OGG1-AP-qPCR at the (**c**) *FOS*, *CCN2*, *SRF* promoters and (**d**) *TE6189* enhancer. Bar charts show fold change in % input relative to the untransfected cells (n=3 biological replicates, mean±SEM for *FOS* promoter and *TE6189*; n=2 biological replicates, range for *CCN2* and *SRF* promoters). Two-sided unpaired Student t-test was conducted. (**e**) Transfected cells were serum starved for 48 hours, then left untreated or treated with 10 µM H_2O_2 for 10 minutes on ice in the dark and recovered in serum-containing media for 90 minutes. Transcript levels were measured by qPCR. Expression in untreated and siSCR H_2O_2-treated cells were normalised to siSCR untreated cells, while all other conditions were normalised to siSCR H_2O_2-treated cells (n=2 biological replicates, range).

Figure S8: Uncropped blots (Related to Figures 1, 3 and S2)

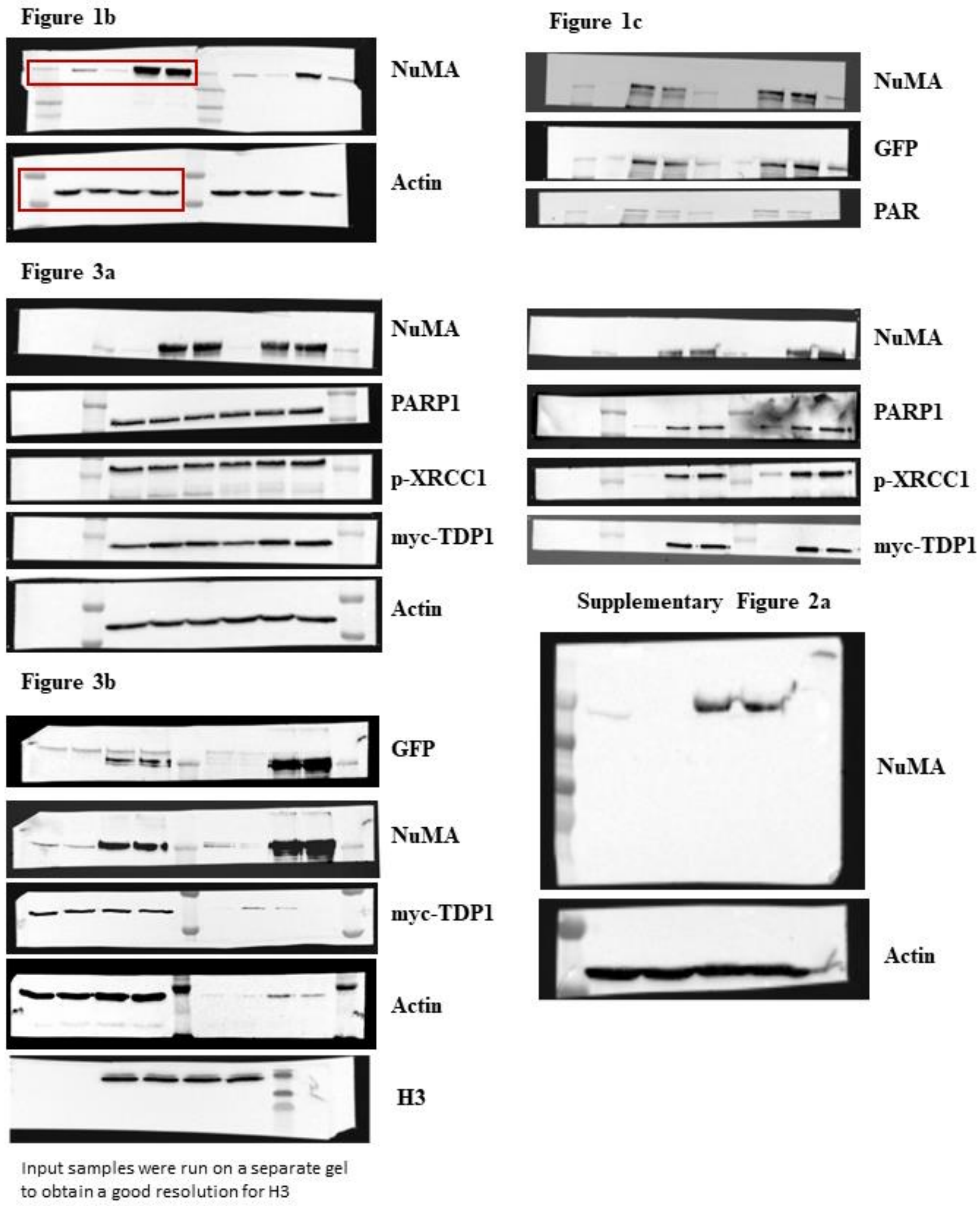

Figure S8: Uncropped blots

Uncropped images of the blots shown in the manuscript.

Table S1: ADP-ribosylation sites that are mutated in NuMA^{PARmut} (related to Figure 2)

Table S1: Amino acid positions that have been mutated in the WT construct to generate the PARmut construct.

Amino Acid Position	Amino acid in NuMA^{WT}	Amino acid in NuMA^{PARmut}
**1609**	H	A
**1806**	S	A
**1840**	S	A
**1852**	S	A
**1887**	S	A
**1892**	S	A
**1969**	S	A
**1991**	S	A
**2003**	S	A
**2008**	S	A
**2009**	C	A
**2047**	S	A
**2051**	S	A
**2062**	S	A
**2069**	S	A
**2082**	S	A

Table S2: List of primers used in this study

Table S2: Sequences of the primers and DNA block used in this study

Primer name	Primer sequence	Purpose	Reference
**1609_Fwd**	CAGAAGGAGCAGGCAGCTGAGGCCTATAAGCTGCAGATGGAG	Generation of NuMA^{PARmut}	This study
**1609_Rev**	CTCCATCTGCAGCTTATAGGCCTCAGCTGCCTGCTCCTTCTG	Generation of NuMA^{PARmut}	This study
**2082_Fwd**	GGCTTCCCCCAACACTCGCGCTGGAACCCGCCGTTCTCCG	Generation of NuMA^{PARmut}	This study
**2082_Rev**	CGGAGAACGGCGGGTTCCAGCGCGAGTGTTGGGGGAAGCC	Generation of NuMA^{PARmut}	This study
**AF**	TCCAAGAGTTGGCAGCCGTACG	Generation of NuMA^{PARmut}	This study
**AR**	GCGGGTCTTACGACCCGAGTC	Generation of NuMA^{PARmut}	This study
**BF**	GGAGACGTCTTCCTGGACTCG	Generation of NuMA^{PARmut}	This study
**BR**	TTATCTAGATCCGGTGGATCCG	Generation of NuMA^{PARmut} & NuMA^{Corrected}	This study
**DNA block**	GGAGACGTCTTCCTGGACTCGGGTCGTAAGACCCGCGCCGCTCGTCGGCGCACCACGCAGATCATCAACATCACCATGACCAAGAAGCTAGATGTGGAAGAGCCAGACAGCGCCAACTCATCGTTCTACAG	Generation of NuMA^{PARmut}	This study

Primer name	Primer sequence	Purpose	Reference
	CACGCGGGCTGCTCCTGCTTCCCAGGC TAGCCTGCGAGCCACCGCCTCTACTCA GTCTCTAGCTCGCCTGGGTTCTCCCGA TTATGGCAACTCAGCCCTGCTCAGCTT GCCTGGCTACCGCCCCACCACTCGCA GTTCTGCTCGTCGTGCCCAGGCCGGGG TGGCCAGTGGGGCCCCTCCAGGAAGG AACAGCTTCTACATGGGCACTTGCCA GGATGAGCCTGAGCAGCTGGATGACT GGAACCGCATTGCAGAGCTGCAGCAG CGCAATCGAGTGTGCCCCCCACATCTG AAGACCTGCTATCCCCTGGAGTCCAG GCCTTCCCTGAGCCTGGGCACCATCAC AGATGAGGAGATGAAAACTGGAGACC CCCAAGAGACCCTGCGCCGAGCCGCC ATGCAGCCAATCCAGATAGCCGAGGG CACTGGCATCACCACCCGGCAGCAGC GCAAACGGGTCGCCCTAGAGCCCCAC CAGGGCCCTGGAACTCCTGAGGCTAA GAAGGCCACCGCCGCTTTCCCACGCC CCATGACTCCCCGAGACCGACATGAA GGGCGCAAACAGAGCACTACTGAGGC CCAGAAGAAAGCAGCTCCAGCTTCTA CTAAACAGGCTGACCGGCGCCAGGCG ATGGCCTTCGCTATCCTCAACACACCC AAGAAGCTAGGGAACGCCCTTCTGCG GCGGGGAGCCGCAAAGAAGGCCCTGT CCAAGGCTTCCCCCAACACTCGCGCT		
**AF2**	TCCAAGAGTTGGCAGCCGTACG	Generation of NuMA^{Corrected}	This study
**1528_Fwd**	GAGAGGCAGCGGTTCCAGGAAGAGAG GCAGAAACTCAC	Generation of NuMA^{Corrected}	This study
**1528_Rev**	GTGAGTTTCTGCCTCTCTTCCTGGAAC CGCTGCCTCTC	Generation of NuMA^{Corrected}	This study
**FOS_TSS_Fwd**	ACACTCATTCATAAAACGCTTGT	AP-qPCR	This study
**FOS_TSS_Rev**	CTGCAGATGCGGTTGGAGTA	AP-qPCR	This study
**SRF_TSS_Fwd**	ACCATATAAGGAGCGGCCTCG	AP-qPCR	This study
**SRF_TSS_Rev**	GGGATCCCCCGACCCTTC	AP-qPCR	This study
**CCN2_TSS_Fwd**	CCCCAAACTCACACAACAACTC	AP-qPCR	This study
**CCN2_TSS_Rev**	GCGGCTGCCGTCGAG	AP-qPCR	This study
**TE1869_Fwd**	ATCAGCCAGTAGATAA	AP-qPCR	This study
**TE1869_Rev**	AGCGACTGGAGTAAGG	AP-qPCR	This study
**FOS_Fwd**	GGAGAATCCGAAGGGAAAGGA	qPCR	This study
**FOS_Rev**	GTTGGTCTGTCTCCGCTTGG	qPCR	This study
**CCN2_Fwd**	CGA GGA GTG GGT GTG TGA C	qPCR	This study
**CCN2_Rev**	TTC CAG TCG GTA AGC CGC	qPCR	This study
**KLF6_Fwd**	GAG GAG TAC TGG CAA CAG ACC T	qPCR	This study
**KLF6_Rev**	GCT GAA ACA TAG CAG GGC TC	qPCR	This study
**JUN_Fwd**	GAG CTG GAG CGC CTG ATA AT	qPCR	This study
**JUN_Rev**	CCC TCC TGC TCA TCT GTC AC	qPCR	This study

Table S3: List of siRNA sequences used in this study

Table S3: siRNA sequences used for transfection

siRNA	siRNA Sequence	References
**siSCR**	5’-AGGUAGUGUAAUCGCCUUG-3’	[1]
**siNuMA 1**	5'-GGUGGCAACUGAUGCUUUA-3'	[1]

References

1. Ray, S., Abugable, A.A., Parker, J., Liversidge, K., Palminha, N.M., Liao, C., Acosta-Martin, A.E., Souza, C.D.S., Jurga, M., Sudbery, I., et al. (2022). A mechanism for oxidative damage repair at gene regulatory elements. Nature *609*, 1038–1047. 10.1038/s41586-022-05217-8.

2. Hudson, J.J.R., Chiang, S.-C., Wells, O.S., Rookyard, C., and El-Khamisy, S.F. (2012). SUMO modification of the neuroprotective protein TDP1 facilitates chromosomal single-strand break repair. Nat Commun *3*, 733. 10.1038/ncomms1739.
